# *Arabidopsis thaliana* alcohol dehydrogenase is differently affected by several redox modifications

**DOI:** 10.1371/journal.pone.0204530

**Published:** 2018-09-25

**Authors:** Sébastien Dumont, Natalia V. Bykova, Alexia Khaou, Yasmine Besserour, Maude Dorval, Jean Rivoal

**Affiliations:** 1 Institut de Recherche en Biologie Végétale, Université de Montréal, Montréal, Québec, Canada; 2 Morden Research and Development Centre, Agriculture and Agri-Food Canada, Morden, Manitoba, Canada; University of Tennessee, UNITED STATES

## Abstract

In plant cells, many stresses, including low oxygen availability, result in a higher production of reactive oxygen species (ROS) and reactive nitrogen species (RNS). These molecules can lead to redox-dependent post-translational modification of proteins Cys residues. Here, we studied the effect of different redox modifications on alcohol dehydrogenase (ADH) from *Arabidopsis thaliana*. ADH catalyzes the last step of the ethanol fermentation pathway used by plants to cope with energy deficiency during hypoxic stress. *Arabidopsis* suspension cell cultures showed decreased ADH activity upon exposure to H_2_O_2_, but not to the thiol oxidizing agent diamide. We purified recombinant ADH and observed a significant decrease in the enzyme activity by treatments with H_2_O_2_ and diethylamine NONOate (DEA/NO). Treatments leading to the formation of a disulfide bond between ADH and glutathione (protein *S*-glutathionylation) had no negative effect on the enzyme activity. LC-MS/MS analysis showed that Cys47 and Cys243 could make a stable disulfide bond with glutathione, suggesting redox sensitivity of these residues. Mutation of ADH Cys47 to Ser caused an almost complete loss of the enzyme activity while the Cys243 to Ser mutant had increased specific activity. Incubation of ADH with NAD^+^ or NADH prevented inhibition of the enzyme by H_2_O_2_ or DEA/NO. These results suggest that binding of ADH with its cofactors may limit availability of Cys residues to redox modifications. Our study demonstrates that ADH from *A*. *thaliana* is subject to different redox modifications. Implications of ADH sensitivity to ROS and RNS during hypoxic stress conditions are discussed.

## Introduction

Reactive oxygen species (ROS) and reactive nitrogen species (RNS) are common by-products of plant cellular metabolism [1]. During stress, the increase of ROS and RNS concentrations can lead to damages of cell macromolecules, including proteins via their Cys thiol groups [2–5]. Spontaneous oxidation of sensitive Cys residues by ROS leads to the formation of sulphenic acid that can further be oxidized to irreversible products such as sulphinic acid and sulphonic acid. Sulphenic acid can also react with a reduced thiol group to form intra- or inter-molecular disulfide bonds, or disulfide bond with reduced glutathione (GSH; protein *S*-glutathionylation). Similarly to ROS, RNS such as nitric oxide (NO) can react with redoxsensitive Cys thiols to form nitrosothiols (-SNO) in the process called protein *S*-nitrosylation [6]. These redox PTMs can be reversed *in vitro* by addition of reductants such as DTT or *in vivo* by specific oxidoreductases such as thioredoxins or glutaredoxins [7, 8].

Hypoxia can occur in plants during flooding but also in normal conditions, during seed development and germination as well as in bulky organs due to limited oxygen diffusion [9, 10]. Paradoxically, several observations reported increased ROS and RNS concentrations as well as induction of genes coding for antioxidant enzymes following hypoxia in plants [11–19]. Alcohol dehydrogenase (ADH, EC 1.1.1.1) is a Zn-binding enzyme that catalyzes the reversible conversion of acetaldehyde to ethanol while oxidizing NADH to NAD^+^ [20]. During hypoxia, it ensures the maintenance of the glycolytic flux by recycling NAD^+^ [21] and controls toxic acetaldehyde produced by the decarboxylation of pyruvate [22]. In *A*. *thaliana*, the single gene encoding ADH [20] was shown to be necessary for survival in hypoxia [22]. Recently, its involvement in the response to other biotic and abiotic stresses was also demonstrated [23]. Several lines of evidence point to the occurrence of ADH redox modifications in different systems [24–29]. Yeast ADH (YADH) activity is known to be inhibited by oxidation with H_2_O_2_ with Zn loss from the enzyme structure [30, 31]. Moreover, YADH and mammallian ADH are inhibited by NO donors leading to a loss of Zn atoms from the protein structure [27, 30, 32].

Although the transcriptional and post-transcriptional regulation of plant ADHs gene expression under hypoxia/anoxia has been thoroughly studied [33–36], little is still known about ADH PTMs in photosynthetic organisms. A first indication that plant ADH activity could be modulated by a redox mechanism came from the report of a redox-dependent inactivator of ADH in rice seedlings [37]. ADH was later found as a putative target of the cytosolic thioredoxin *h* in *A*. *thaliana*, further suggesting redox sensitivity [38]. ADH was also identified as a target of protein *S-*glutathionylation in a survey of *A*. *thaliana* cell extracts [24]. Moreover, two proteomic studies showed that ADH was *S-*nitrosylated *in vivo* in *A*. *thaliana* suspension cell cultures and *Solanum tuberosum* tubers [25, 29]. Despite several indications of redox modification on *A*. *thaliana* ADH, very little is known about the effects of these modifications on its enzymatic activity. Considering the existing literature on ADH, we hypothesized that *A*.*thaliana* ADH activity could be sensitive to different redox modifications via its Cys residues. We also hypothesized that different types of redox modifications could have different effects in term of reversibility and alteration of ADH enzymatic activity.

Here, we show that oxidative stress generated by H_2_O_2_ treatment results in a decrease in ADH activity in *A*. *thaliana* cells. We purified recombinant ADH and measured its inactivation by H_2_O_2_ and DEA/NO. We identified two redox active Cys residues that can form disulfide bonds with glutathione. However, treatments inducing *S-*glutathionylation did not decrease enzyme activity. Incubation with NAD^+^ and NADH reduced inhibition of the enzyme by H_2_O_2_ and DEA/NO. Taking these results together, we propose that *A*. *thaliana* ADH is sensitive to inhibition by ROS-driven protein oxidation or RNS-mediated *S*-nitrosylation.

## Materials and methods

### Chemicals

Except when indicated otherwise, buffers, chemicals, and reagents were of analytical grade from Sigma Chemical Co. or Thermo Fisher Scientific. Biotinylated glutathione ethyl ester (BioGEE) and biotinylated GSSG (BioGSSG) were synthesized as described [39].

### Culture and oxidative treatment of *Arabidopsis thaliana* cells

*A*. *thaliana* suspension culture cell line PSB-D was obtained from the *Arabidopsis* Biological Resource Center (ABRC). Cells were grown at 22°C in the dark with shaking at 140 RPM and subcultured every 14 days in Murashige and Skoog medium [40] containing 3% (w/v) sucrose. For oxidative treatments, cells were used at day 7 (mid-log growth phase) of the culture cycle. Cell culture aliquots (10.5 ml, ~350 mg cell fresh weight (FW)) were incubated for 30 min with 10 mM H_2_O_2_ or 4.5 mM diamide (H_2_O was added for controls). Cells were then harvested by filtration, quickly washed with 4 ml of 100 mM Tris-Cl pH 7.5, flash frozen in liquid N_2_ and stored at -80°C. Proteins were extracted in 20 mM Tris-HCl pH 7.5, 150 mM NaCl, 5% (v/v) glycerol, 10 mM EDTA, 0.1% (v/v) TritonX-100, 0.5% (w/v) Na deoxycholate, 1 mM Na_3_VO_4_ with a polytron homogenizer (Brinkmann, Mississauga, ON, Canada) with 3×10 sec bursts at maximum power using 1 ml buffer/100 mg FW. After centrifugation at 12,000 x *g* for 30 min at 4°C, the supernatant was used to measure enzyme activities, free thiols and protein concentration.

### Plasmid constructions and site directed mutagenesis

Standard techniques were used for recombinant DNA manipulations [41]. *A*. *thaliana* sequence for wild type (WT) ADH1 (At1g77120) in pUNI51 was obtained from ABRC (stock number: U12940; https://www.arabidopsis.org/abrc/) and amplified by PCR using the following primers: ADH-F1 5’ATGTCTACCACCGGACAGATT3’ and ADH-R1 5’ TGTTAGCAGCCGGATCTTCTA 3’. The resulting amplicon was digested with *Not*I and cloned into the expression vector pProEx HTb (Invitrogen Canada Inc., Burlington, ON, Canada) previously prepared with *Ehe*I and *Not*I. The ligated plasmid was used to transform competent *Escherichia coli* cells (HB101 strain). The C47S and C243S mutants were generated by site directed mutagenesis by NorClone (London, ON, Canada) using pProEx HTb containing the ADH insert. All the constructions used in this study were confirmed by full sequencing.

### Production and purification of recombinant ADH

Recombinant WT and mutant ADH were expressed in *E*. *coli* as previously described [39, 42]. Induction was done overnight at room temperature with 0.6 mM isopropyl β-D-thiogalactoside. Recombinant ADHs were purified under native conditions according to the manufacturer’s instructions (Invitrogen Canada, Burlington, ON, Canada). Purified fractions were pooled and dialyzed against a buffer containing 25 mM Tris-Cl pH 7.5, 1 mM MgCl_2_ and 10 mM DTT. Removal of DTT was done by dialyzing twice against the same buffer containing no DTT. The enzyme preparation was stored at −20°C in 50% (v/v) glycerol and were stable for over 3 months without any significant loss of activity.

### Assays for ADH and GAPDH activities, free thiols, Zn quantification and protein concentration

All enzymatic and colorimetric assays were performed on a VERSAmax or a SpectraMax i3X microplate reader (Molecular Devices, San Diego, CA, USA). ADH activity was measured in the ethanol-to-acetaldehyde direction as described [43]. For determination of *K*_m_ and *k*_cat_ values, we used varying concentrations of NAD^+^ (0–0.8 mM) in the presence of 5 mM DTT. Kinetic parameters were determined using a non-linear regression analysis software (SigmaPlot 8.0, SPSS Inc., Chicago, IL, USA). NAD-GAPDH was measured as described [44] except that DTT was not added to the reaction mixture. Free thiols were measured at 412 nm using DTNB [45] and GSH as standard. Quantification of Zn release was done at 500 nm using 125 μM 4-(2-Pyridylazo)resorcinol and ZnSO_4_ as standard [46]. Recombinant ADH (0.5 nmol) was incubated for one hour with 1 mM H_2_O_2_, 1 mM DEA/NO or 1 mM GSSG in a final volume of 250 μl. Protein were measured by the method of Bradford or the DC (BioRad) method using BSA as a standard [47].

### Oxidative treatment of recombinant ADH

Purified recombinant ADH (4 μg/ml) was incubated at room temperature with 100 mM Tris-Cl pH 7.5 and 0.1–0.75 mM H_2_O_2_, 0.1–1 mM DEA/NO or 0.5–5 mM GSSG. An aliquot of the different samples was taken at different time points for ADH activity. For inhibition of WT and C243S, the same experiment was performed as described above, but only using 0.5 mM of H_2_O_2_ or 0.5 mM of DEA/NO. For reactivation experiments, recombinant ADH was incubated with 100 mM Tris-Cl pH 7.5 and 0.5 mM DEA/NO for 30 min or 0.5 mM H_2_O_2_ for 30 or 60 min. DTT (10 mM final concentration) was then added and ADH activity was measured after 30 min. For diamide + GSH experiments, ADH was incubated with 100 mM Tris-Cl pH 7.5 and 0.5 mM GSH. Diamide (0–0.5 mM) was added in the different samples and ADH activity was measured within 5 min of incubation. For protection experiments with NAD^+^ or NADH, recombinant ADH was incubated with 100 mM Tris-Cl pH 7.5 and 0–2 mM NAD^+^, 0–0.2 mM NADH or 20 mM ethanol. H_2_O_2_ (0.5 mM) or DEA/NO (0.5 mM) was then added and an aliquot of the different samples was taken at different time points for ADH activity.

### Electrophoresis, immunoblotting and immunodetection

SDS-PAGE analysis on 12% (w/v) polyacrylamide gels and immunoblotting were performed as described before [39]. Immunodetection of ADH was carried out with a rabbit anti-ADH polyclonal immune serum (1/1,000 dilution) [48]. The detection was done using a goat anti-rabbit IgG conjugated to alkaline phosphatase secondary antibody (1/10,000 dilution) (Promega, Nepean, ON, Canada). Detection of BioGEE was performed as previously described [39].

### BioGSSG and BioGEE labeling of recombinant proteins

For BioGSSG labelling, recombinant ADH (25 μg/ml) was incubated with 100 mM Tris-Cl pH 7.5 and 0.5 mM BioGSSG. An aliquot was withdrawn at different incubation times and blocked with 100 mM (final concentration) iodoacetamide (IAM) for 30 min. A control was also made in the presence of 20 mM DTT. For BioGEE + diamide experiments, ADH (25 μg/ml) was incubated with 100 mM Tris-Cl pH 7.5 and 0.5 mM BioGEE. The ADH/BioGEE sample was then aliquoted and incubated 5 min with various concentrations of diamide (0–0.5 mM). The reaction was stopped by adding 100 mM IAM for 30 min. All samples were denatured for 5 min at 94°C before immunoblot analysis.

### Biotin switch

The biotin switch protocol was adapted from [49]. ADH (165 μg/ml) was incubated with 100 mM Tris-Cl pH 7.5 and 1 mM DEA/NO for 15 min at room temperature. The reaction was stopped using 4 volumes of cold acetone (-20°C) to the samples during 30 min followed by centrifugation at 4°C for 5 min at 12,000 x *g*. The resulting pellet was resuspended in TENS buffer (30 mM Tris-HCl pH 7.8, 1 mM EDTA, 100 mM NaCl, and 1% SDS) with the addition of 20 mM IAM and 0.1 mM neocuproine. After incubation for 30 min with 2 x 5 min vortexing, proteins were acetone-precipitated again and resuspended in TENS buffer supplemented or not with 10 mM sodium ascorbate (ASC). After incubating 15 min (with 5 min vortexing), free thiols were labeled with monobromobimane (mBBr) which was directly added in the samples at 1 mM final concentration followed by 60 min incubation. Samples were then precipitated and resuspended in TENS buffer. A control was performed without ascorbate to reveal the background for Cys blocking efficiency. Another control was done without DEA/NO treatment to show the background due to the reductive effect of ascorbate on Cys residue(s) not available for the blocking process (e.g., disulfide bonds). Protein concentration was measured using the *DC* Protein Assay and sample buffer was added. After SDS-PAGE followed by visualization with a UV transilluminator, the gel was stained with Coomassie blue.

### Fluorescence emission spectra

Fluorescence was determined using a Molecular Devices SpectraMax i3X microplate reader. Recombinant ADH WT and mutants were diluted (0.1 mg/ml) in 100 mM Tris-Cl pH 7.5. Fluorescence emission spectra were recorded using 20 μg of enzyme with an excitation wavelength of 274 nm.

### NanoLC-MS/MS analysis of modified Cys residues

In-solution trypsin digestion and nano-LC-MS/MS analysis of *in vitro* redox modifications of recombinant ADH were done essentially as described previously [39] using 100 pmol ADH incubated with 2.5 mM GSSG or left untreated (control). Automated nano-flow LC/MS/MS analysis of peptide digests was done under previously described conditions [39] using LTQ XL ion trap mass spectrometer (Thermo Fisher Scientific, San Jose, CA) connected on-line with nano-HPLC (Dionex UltiMate 3000). Data-dependent analysis was performed as described before [39]. Normalized collision energy was set to 35% and the source temperature to 200°C.

Peptide CID fragmentation spectra were searched using Mascot v. 2.4 (Matrix Science, London, UK) against the NCBInr *A*. *thaliana* protein database using a general ID search followed by the Error Tolerant search to allow for protein modification screening. The Mascot MS/MS Ion Search parameters were as follows: (1) tryptic digest with maximum one missed cleavage; (2) monoisotopic peptide masses were used; (3) the peptide mass tolerance was kept at 2 Da; and the fragment ions mass tolerance was set at 0.8 Da; (4) variable modifications Glutathione (C), Carbamidomethyl (C), Pyro-carbamidomethyl (N-term C), Oxidation (M), and Deamidation (NQ) were used; (5) peptide charge state +1, +2 and +3 for LC-MS/MS spectra. Spectra with modifications were verified manually using the GPMAW 9.2 (Lighthouse Data, Odense, Denmark) software.

### Replication and statistical analysis

Data were analyzed using the unpaired Student’s *t*-test tool of SigmaPlot 8.0, with *P* < 0.05 considered as a significant difference. All graphs and figures show representative data of three to five independent experiments. For enzyme activity assays, each independent experiment was the average of at least three technical replicates. Five different protein purifications were produced for experiments with WT ADH. Two different protein purifications were done to perform experiments with ADH mutants. Low variations between purification batches were observed (<10% variation in specific activity).

## Results

### Oxidative treatments of *A*. *thaliana* cell cultures

**Fig 1:**
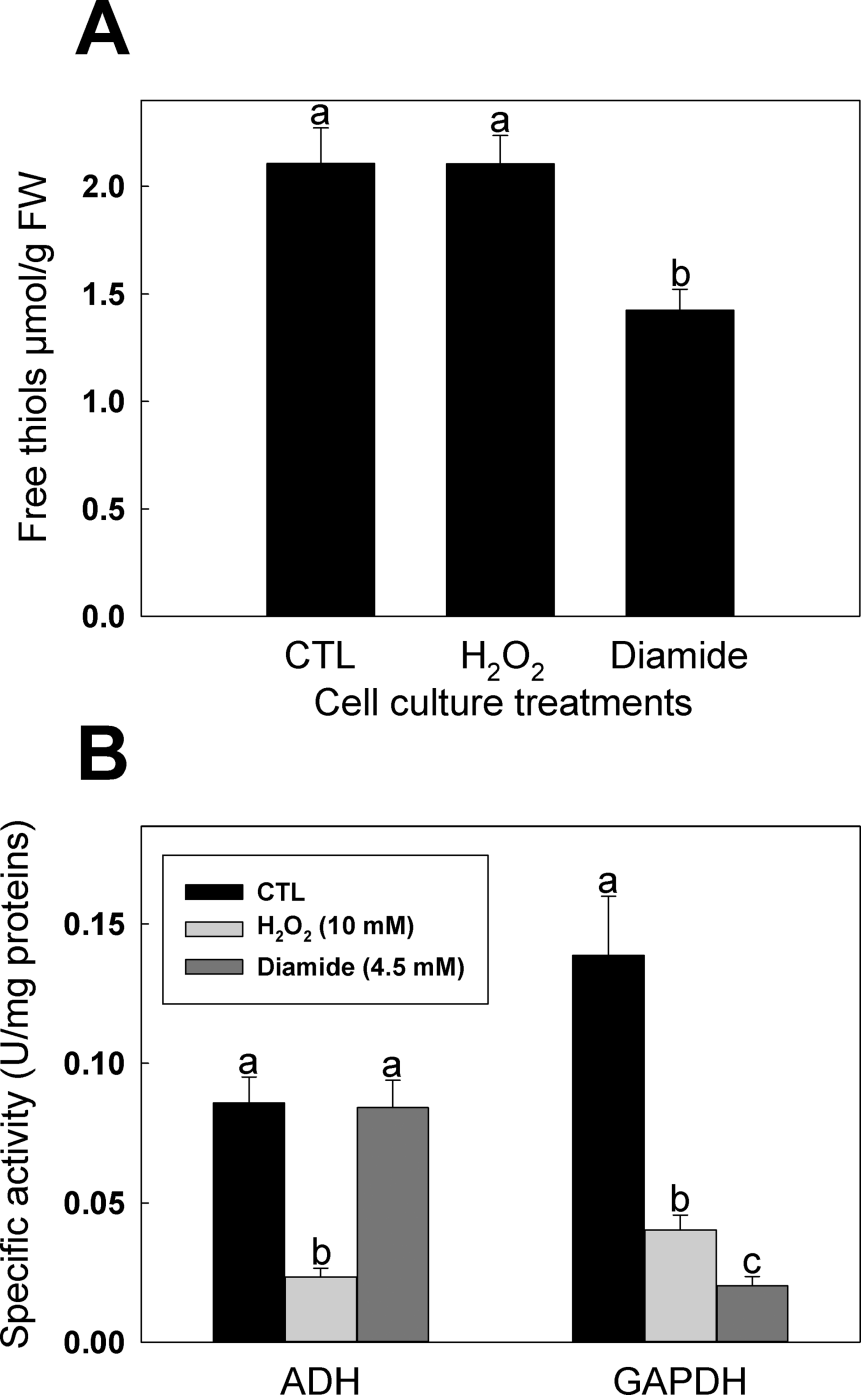
Effects of oxidative treatments on free thiols and enzyme activities in *A*. *thaliana* suspension cell cultures. Cells were incubated with 10 mM H_2_O_2_ or 4.5 mM diamide for 30 min. (**A**) Free thiol content measured by DTNB, (**B**) ADH and GAPDH specific activity in cell extracts after oxidative treatments. Data with different letters are significantly different (Student’s *t*-test, *P* < 0.05).

To study effect of oxidative stress on ADH *in vivo*, *A*. *thaliana* cell cultures were treated with 10 mM H_2_O_2_, 4.5 mM diamide or H_2_O (control). Free thiols content, ADH and GAPDH activities were measured on cell extract from each treatment. H_2_O_2_ treatment did not affect total free thiols in cell extracts (Fig 1A). However, incubation of cells with diamide caused a significant decrease in free thiols compared to the control and H_2_O_2_ treatment (Fig 1A). Diamide is a thiol-specific oxidant that has been used to promote rapid *S*-glutathionylation *in vivo* and *in vitro* [39, 50]. We measured GAPDH activity because this enzyme has been extensively studied for its sensitivity to several redox modifications such as oxidation, *S-*glutathionylation and *S*-nitrosylation [49, 51, 52] and could thus be used to confirm the impact of the oxidative treatments *in vivo*. GAPDH specific activity was significantly decreased by both H_2_O_2_ and diamide treatments compared to the control (Fig 1B). The inhibitory effect of diamide treatment on GAPDH activity in cell extracts was also significantly more important than the effect of H_2_O_2_. In the case of ADH, the enzyme activity in cell extracts was significantly decreased by addition of H_2_O_2_ to the cell culture. However, the diamide treatment did not affect ADH activity (Fig 1B).

### Purification of recombinant ADH from *A*. *thaliana* and sensitivity to H_2_O_2_ and DEA/NO

To further characterize redox modifications on ADH, we expressed recombinant His-tagged ADH from *A*. *thaliana* and purified it from *E*. *coli*. Analysis of the purified protein by SDS-PAGE under reducing conditions showed a single band near the 45 kDa MW standard, close to the expected MW of 50.75 kDa (Figure A in S1 File). In preliminary tests, we observed that ADH activity decreased over time upon dilution without reductant (Figure B in S1 File). We made an independent untreated control sample as a reference to calculate the difference between treated and untreated samples (expressed as percentage of control activity). We thus measured only the difference in the enzyme activity due to the specific oxidative treatment.

**Fig 2:**
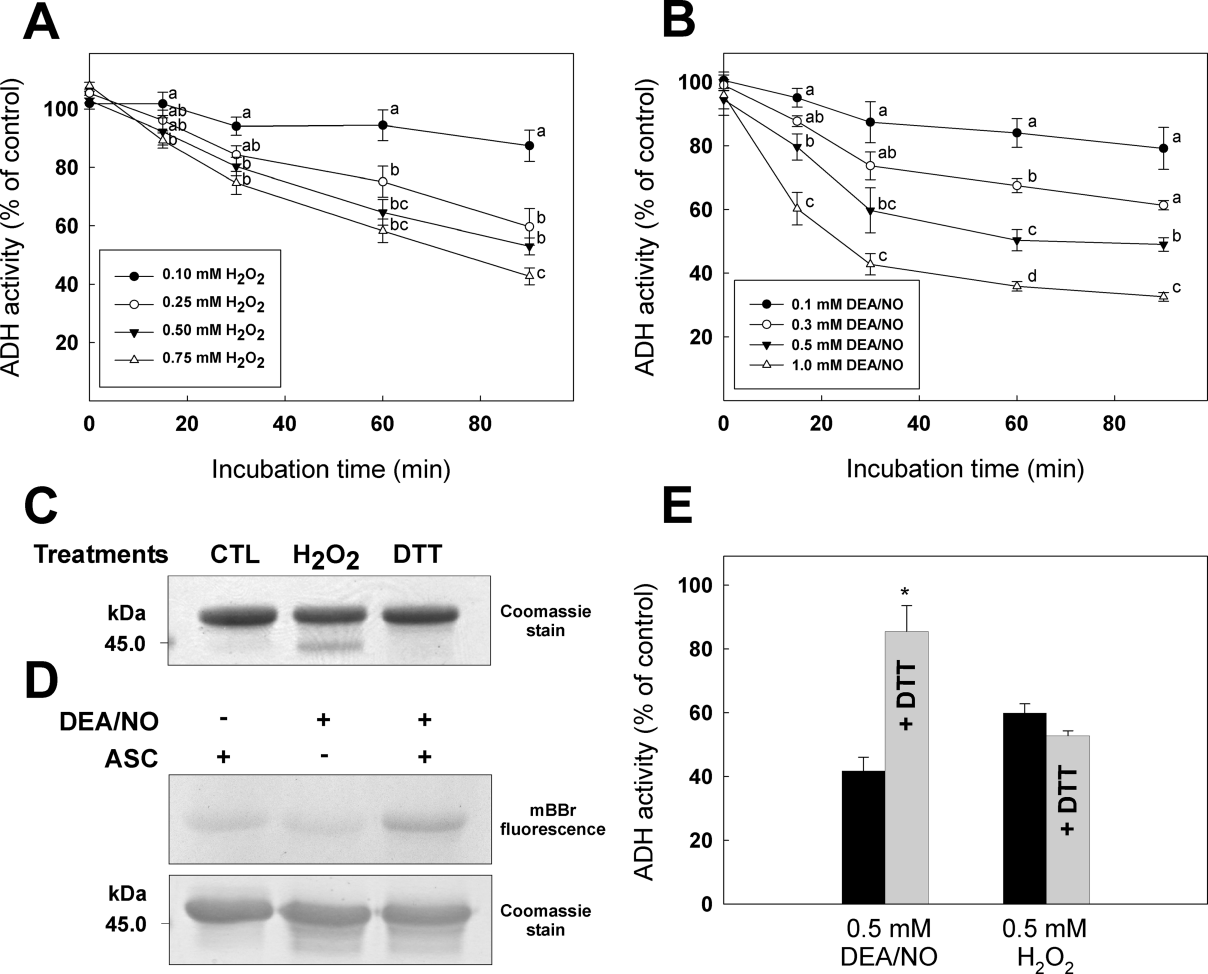
Analysis of the effects of oxidative treatments on *A*. *thaliana* ADH. Inhibition of ADH activity by different concentrations of (**A**) H_2_O_2_ or (**B**) DEA/NO. (**C**) SDS-PAGE analysis of ADH treated with 0.5 mM H_2_O_2_ or 10 mM DTT. (**D**) Analysis of ADH by the biotin-switch technique after treatment with 1 mM DEA/NO (Upper panel, mBBr fluorescence; Lower panel, Coomassie staining). (**E**) Reactivation of ADH by DTT. ADH was inhibited by 0.5 mM DEA/NO for 30 min or 0.5 mM H_2_O_2_ for 60 min, 10 mM DTT was then added in the sample. Data with different letters or the symbol (*) indicate significant differences (Student’s *t*-test, *P* < 0.05).

Recombinant ADH was then used to study sensitivity to oxidation and *S-*nitrosylation (Fig 2). Incubation of ADH with various concentrations (0.1–0.75 mM) of H_2_O_2_ caused a decrease of the enzyme activity (Fig 2A). Similarly, treatment with DEA/NO (0.1–1 mM) decreased ADH activity (Fig 2B). Treatment of ADH with H_2_O_2_ led to the formation of a higher mobility band on non-reductive SDS-PAGE (Fig 2C**)**. This band was not observed in the CTL or DTT-treated samples. The occurrence of higher mobility bands on non-reductive SDS-PAGE following oxidative treatment suggests the formation of intramolecular disulfide bonds [53].We also used the biotin-switch technique to show the presence of nitrosothiol(s) on recombinant ADH [54] (Fig 2D). In Fig 2D (upper panel), the mBBr fluorescence for the *S-*nitrosylated ADH sample treated with ascorbate is distinctly higher than the background in both control samples. Gel staining with Coomassie blue performed after exposure to UV (Fig 2D, lower panel) demonstrates equal loading of the samples. It is also possible to see that both samples treated with DEA/NO show an altered migration pattern (presence of higher mobility bands).

After decreasing ADH activity by using DEA/NO or H_2_O_2_, we added DTT in an attempt to reverse the effect of oxidative treatments (Fig 2E). Addition of DTT after treatment with DEA/NO allows ADH to recover a great part of its enzymatic activity. However, addition of DTT after inhibition by H_2_O_2_ did not restore ADH activity. Sulphenic acid and disulfide bonds are usually reduced by DTT, however, further oxidation of sulphenic acid to sulphinic acid and sulphonic acid are not reversible by reductants. Results obtained in Fig 2E suggest that Cys residues responsible for ADH inhibition by H_2_O_2_ are oxidized to irreversible forms. In Fig 2E ADH was incubated with H_2_O_2_ for 60 min before addition of DTT. Addition of DTT after a shorter incubation time (30 min) was still unable to recover ADH activity in the samples (Figure C in S1 File).

### *S*-glutathionylation of recombinant ADH

**Fig 3:**
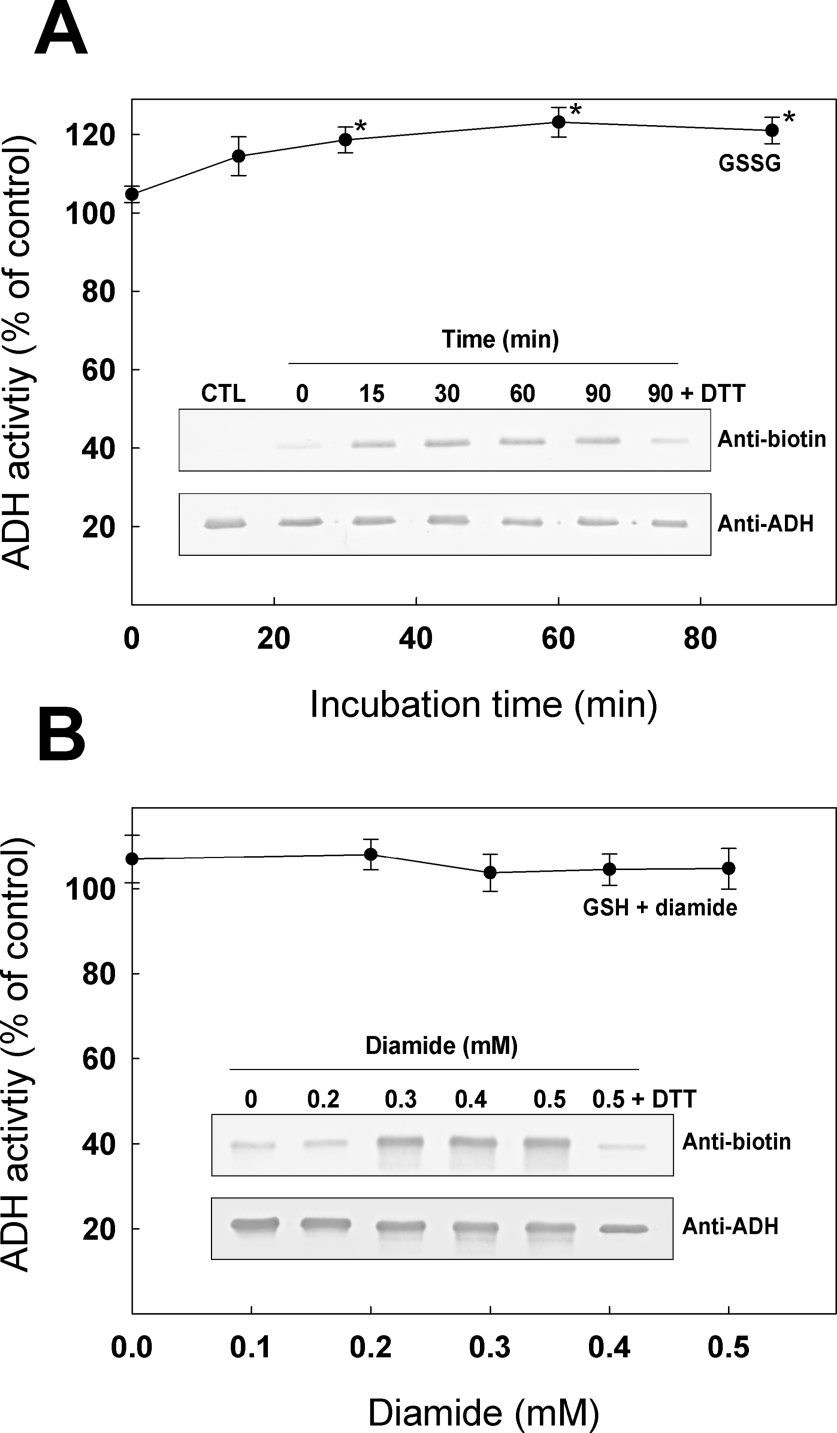
Modification of ADH by *S*-glutathionylation. (**A**) ADH was incubated with 0.5 mM GSSG and activity was measured at various incubation time. **Insert**: ADH was incubated with 0.5 mM BioGSSG for various incubation time and analysed by western blot (upper panel, α-biotin detection; lower panel, α-ADH detection). (**B**) ADH was incubated with 0.5 mM GSH and activity was measured after 5 min of incubation with various diamide concentrations. **Insert**: ADH was incubated with 0.5 mM BioGEE with varying concentration of diamide and analysed by western blot (upper panel, α-biotin detection; lower pane, α-ADH detection). The symbol (*) indicates a significant difference with time 0.

We tested recombinant ADH for its sensibility to undergo *S*-glutathionylation and the possible effects of this modification on the enzyme activity (Fig 3). Oxidized glutathione (GSSG) has been known and widely used to promote *S*-glutathionylation *in vitro* by thiol disulfide exchange [55]. Here, we incubated ADH with 0.5 mM of GSSG for 90 min (Fig 3A). Recombinant ADH activity did not decrease upon incubation with GSSG. In contrast, ADH activity was more stable over time when incubated with GSSG. This increase in the enzyme stability led to an increase (~20%) in activity compared to the control (significant after 30 min). Western blot analyses in Fig 3A show streptavidin detection (upper panel) and α-ADH detection (lower panel) of the recombinant enzyme incubated with BioGSSG. After 15 min of incubation with BioGSSG, a band is clearly visible, showing *S*-glutathionylation of recombinant ADH. Addition of DTT after 90 min of incubation with BioGSSG removed a large part of the *S*-glutathionylation signal detected by streptavidin. We also incubated recombinant ADH with 0.5 mM GSH and different concentrations of diamide (Fig 3B). Here, we found that ADH activity was not significantly affected by diamide + GSH treatment. However, treatment of ADH with BioGEE in the presence of diamide allowed us to detect *S*-glutathionylation of the enzyme on western blot (Fig 3B, insert). The upper panel shows that streptavidin detection of ADH led to a strong signal on nitrocellulose membrane when the enzyme was incubated with 0.5 mM BioGEE and 0.3 to 0.5 mM diamide. When ADH was incubated with 0.5 mM BioGEE and 0.5 mM diamide but in the presence of DTT, the *S*-glutathionylation signal on ADH was reduced to the background level showing the reversibility of the modification.

We measured Zn release in ADH samples treated with GSSG to see if oxidative treatments can affect ADH Zn binding properties. We also tested Zn release for treatment with H_2_O_2_ and DEA/NO (Figure D in S1 File). For each treatment, the background value for Zn release (ADH without treatment) was subtracted from the measured value. Treatment with GSSG did not significantly promote the release of Zn from recombinant ADH. However, Zn release was observed for H_2_O_2_ and DEA/NO treatments and was significantly higher than for controls.

### Identification of *S*-glutathionylated residues in ADH peptides

**Fig 4:**
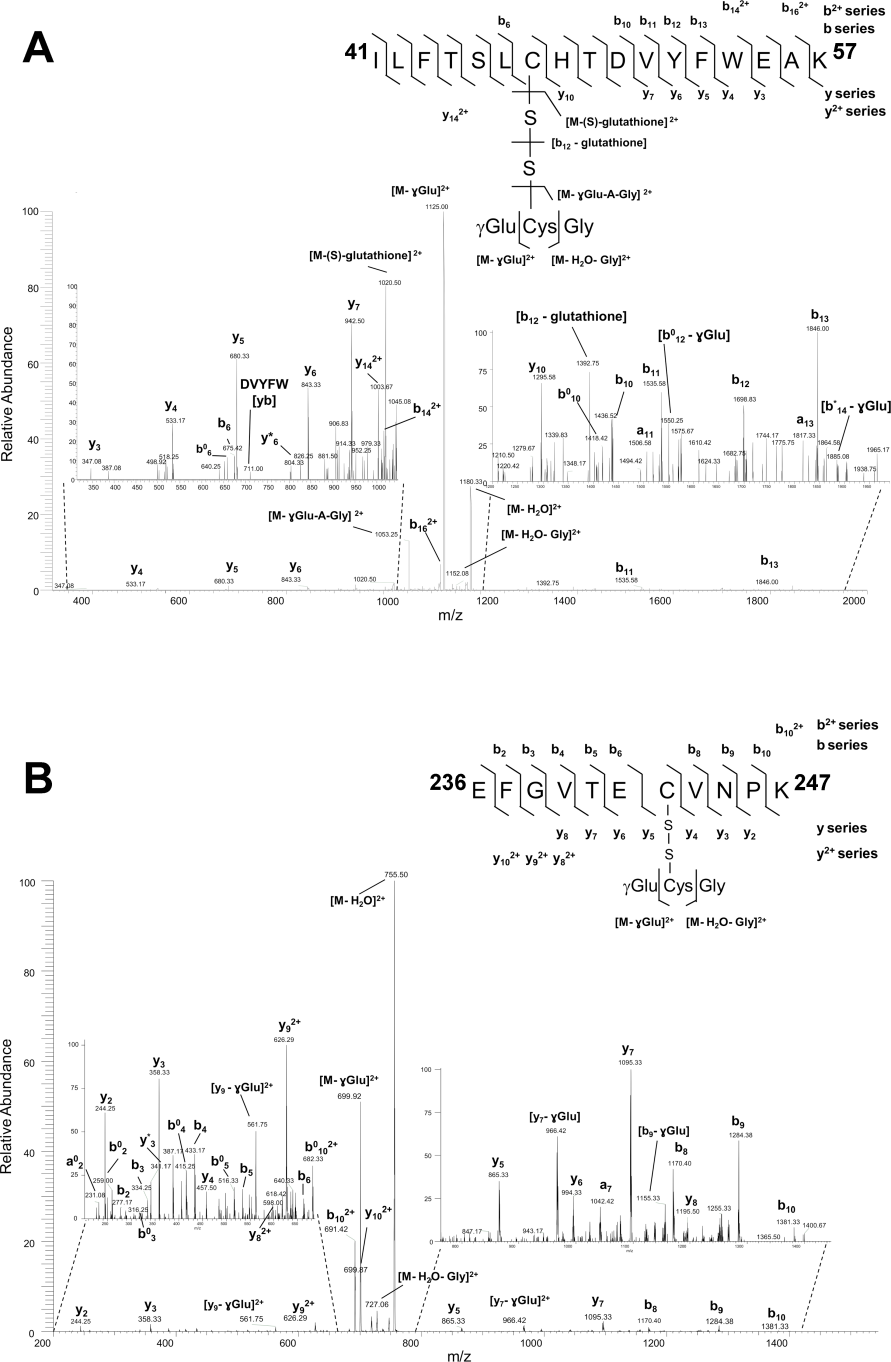
NanoLC-MS/MS analysis of ADH tryptic peptides modified by GSSG. (**A**) CID MS/MS fragmentation spectrum of the precursor ion at *m/z* of 1189.62^2+^ corresponding to a glutathionylated peptide with the 17 amino acids long sequence ^41^ILFTSLCHTDVYFWEAK^57^ (Cys47). The detection of ion at *m/z* 1180.33 indicated the loss of water (-18 Da) resulting in the ion [M—H_2_O]^2+^, while the concomitant loss of Gly (-57 Da) resulted in the ion at *m/z* of 1152.08^2+^ corresponding to [M—H_2_O - Gly]^2+^. The loss of ɣGlu (-129 Da) from the doubly protonated precursor ion resulted in the ion at *m/z* of 1125.00^2+^ corresponding to [M - ɣGlu]^2+^. In [M- ɣGlu-A-Gly] ^2+^ A stands for alanine residue that is formed from Cys in glutathione peptide via radical-based desulfurization mechanism (delta 32 Da), whereas in the main peptide Cys residue is converted into dehydroalanine residue via the β-elimination mechanism (delta 34 Da). In [b12 –glutathione] cysteinyl is a radical. (**B**) CID MS/MS fragmentation spectrum of the precursor ion at *m/z* of 764.40^2+^ corresponding to a glutathionylated peptide with the 11 amino acids long sequence ^237^EFGVTECVNPK^247^ (Cys243). The detection of ion at *m/z* 755.50 indicated the loss of water (-18 Da) resulting in the ion [M—H_2_O]^2+^, while the concomitant loss of Gly (-57 Da) resulted in the ion at *m/z* of 727.06^2+^ corresponding to [M—H_2_O - Gly]^2+^. The loss of ɣGlu (-129 Da) from the doubly protonated precursor ion resulted in the ion at *m/z* of 699.92^2+^ corresponding to [M - ɣGlu]^2+^. The expanded regions of the product ion spectra showing detailed fragmentation pathways of *S*-glutathionylated peptides are given on both left and right sides of the panels A and B. Sequence specific y-, a- and b-type fragment ion signals of different charge states (+1 and +2) identifying the peptide, and decomposition product ions resulting from further internal fragmentation of glutathione adduct by elimination of ɣGlu or Gly residues are indicated. The peaks denoted y^0^/ b^0^ and y*/ b* are the result of water (-18 Da) or ammonia (-17 Da) loss from the corresponding ion, respectively. Product maps indicating the cleavage sites in CID MS/MS are given on the right side of panels **A** and **B**.

*S*-glutathionylation of ADH was induced by incubation with GSSG. *S*-glutathionylated ADH was digested with trypsin and analyzed by nanoLC-MS/MS. Fig 4A shows the collision-induced dissociation (CID) spectrum of the doubly charged precursor ion corresponding to the S-glutathionylated peptide ^41^ILFTSLCHTDVYFWEAK^57^ (*m/z* = 1189.62^2+^). This peptide contains Cys47 and the fragmentation pattern in the CID spectrum shows the presence of ions characteristic of a disulfide bond cleavage between this Cys residue and glutathione. The product ions at m/z of 1020.50^2+^ and high intensity ion at m/z of 1053.25^2+^ correspond to both symmetric and non-symmetric -S-S- bridge cleavage resulting in [M-(S)-glutathione]^2+^ and [M-ɣGlu-A-Gly]^2+^, respectively. In the main peptide the Cys residue is converted into dehydroalanine via the β-elimination mechanism (delta 34 Da), whereas the second Cys residue in glutathione tripeptide is converted into alanine residue formed via radical-based desulfurization mechanism (delta 32 Da) in enthalpically favored non-symmetric cleavage with retention of sulfurs (Fig 4A). The CID spectrum of the doubly charged precursor ion corresponding to the *S*-glutathionylated peptide with sequence ^236^EFGVTECVNPK^247^ (*m/z* = 764.40^2+^) is shown in Fig 4B. The peptide contains Cys243 and *S*-glutathionylation on this residue is confirmed in the CID spectrum. Both ions corresponding to *S-*glutathionylated ADH peptides were not found in samples that had been reduced with DTT and alkylated with IAM. In the CID spectra, the presence of dominant product ions corresponding to the loss of ɣ-glutamyl residue [M-ɣGlu]^2+^ from the glutathione moiety of *S*-glutathionylated precursor peptides ^41^ILFTSLCHTDVYFWEAK^57^ (resulting *m/z* = 1125.00^2+^) and ^236^EFGVTECVNPK^247^ (resulting *m/z* = 699.92^2+^), characteristic fragments of glutathione *S*-conjugates, further corroborates the modification on these peptides. Moreover, the elimination of the glycine moiety due to peptide-bonded glutathione fragmentation was detected for both peptides and resulted in [M-Gly]^2+^ product ion for ^41^ILFTSLCHTDVYFWEAK^57^ (*m/z* = 1152.08^2+^) peptide and [M—H_2_O - Gly]^2+^ ion for ^236^EFGVTECVNPK^247^ (*m/z* = 727.06^2+^) peptide.

By aligning the ADH sequence from *A*. *thaliana* with two yeast ADHs **(**Figure E in S1 File), we can observe that Cys47 corresponds to the yeast Cys43 residues previously shown to be redox-modified [26, 31]. ADHs from *S*. *cerevisiae* and *K*. *lactis* do not have a Cys residue corresponding to Cys243. We also aligned ADH sequences form different photosynthetic organisms (Figure F in S1 File) to demonstrate that Cys47 is strictly conserved between photosynthetic organisms whereas Cys243 is only present in *A*. *thaliana* sequence.

### ADH presence of intrachain disulfide bonds

The comparison of tryptic digests of GSSG-treated and untreated native recombinant ADH and denatured protein (reduction and heating with DTT, followed by IAM alkylation prior to digestion), was also used to explore the redox state of other Cys residues and the positions of disulfide bonds essential for protein structure. The nanoLC-MS/MS analysis allowed for detection of three intramolecular thiol-disulfide bonds in both GSSG-treated and untreated native ADH protein digests (Figures G and H in S1 File). Six Cys residues were found to be involved in the intrachain disulfide bond formation Cys99, Cys102, Cys105, Cys 113, Cys173, and Cys177. A peptide with the sequence ^66^IFGHEAGGIVESVGEGVTDLQPGDHVLPIFTGECGECR^103^ containing an -S-S- bond between Cys99 and Cys102 was detected in doubly- and triply-protonated precursor ion forms at *m/z* of 1962.067^2+^ and 1308.52^3+^, respectively, which is consistent with the calculated *m/z* values for the peptide with dehydro Cys residues. A series of y-type ions containing the intact intrachain disulfide bond were identified with the mass difference of 2 Da in two oxidised dehydro Cys residues attributed to the presence of cystine residue (Figure G in S1 File). The detection of high intensity ion at *m/z* 1930.17^2+^ indicates the loss of perthiyl radical (-SS▪; -64 Da) from the doubly charged precursor resulting in the ion [M—SS▪]^2+^ with the original cysteine residue modified to dehydroalanine. Furthermore, the isotopically resolved peaks indicating ions charge state showed characteristic signature yn + 1 Da (n = 12–14, 17) ions in higher abundance than their corresponding y ions, resulting from the intramolecular hydrogen transfer reactions to the thiyl radical from Val and/or Leu residues (Figure G, panel A in S1 File). Experimental and theoretical studies have shown that intramolecular hydrogen transfer to the thiyl radical is a facile process within peptides and cysteine ions [56, 57], which in our results was apparent under CID conditions in doubly protonated but not in higher charge state precursor ions. An extensive peptide backbone and internal fragmentation in triply protonated precursor ion under CID conditions allowed for detection of the ion at m/z of 506.33^+^ corresponding to the internal fragment with the intact disulfide bond and the loss of 59 Da (CH_5_N_3_) from Arg side-chain. Two other intrachain disulfide bond-bearing peptide ions corresponding to the sequences ^104^HCHSEESNMCDLLR^117^ and ^172^VCIVSCGLSTGLGATLNVAK^191^ were detected at m/z of 557.62^3+^ and 952.57^2+^, respectively. The product ion spectra of both peptides contained series of b-type ions with the delta mass difference of 2 Da indicating the intact intrachain disulfide bond and two oxidised dehydro Cys residues in the corresponding peptide (Figure H, panels A and B in S1 File). Higher internal fragmentation was evident for the triply-protonated ion (peptide ^104^HCHSEESNMCDLLR^117^) with signature neutral loss of 33 Da (•SH) due to the cleavage at the disulfide bond with subsequent elimination of •SH moiety to form dehydroalanine, which resulted in high abundance ion at *m/z* 546.58^3+^ corresponding to [M—SH]^3+^ (Figure H, panel A in S1 File). In addition, y-type ions with one Cys residue being modified to a sulfhydryl group (SH) as a result of the disulfide bond cleavage were also present in the CID spectra. Fragmentation of the doubly charged peptide ^172^VCIVSCGLSTGLGATLNVAK^191^ did not show any b- or y-type fragment ions resulting from the cleavage of peptide bonds between two linked Cys residues indicating lower CID fragmentation efficiency for both an amide linkage and the disulfide bond (Figure H, panel B in S1 File).

Our results suggest that six Cys residues are present in the oxidized disulfide bond form independently of glutathionylation treatment. Among the six Cys residues, Cys99, Cys102, Cys105, and Cys 113 are bound to the same structural Zn atom, and Cys 177 is bound to the Zn atom at the catalytic center.

### NAD^+^ and NADH binding to ADH reduce enzyme sensitivity to H_2_O_2_ and DEA/NO

**Fig 5:**
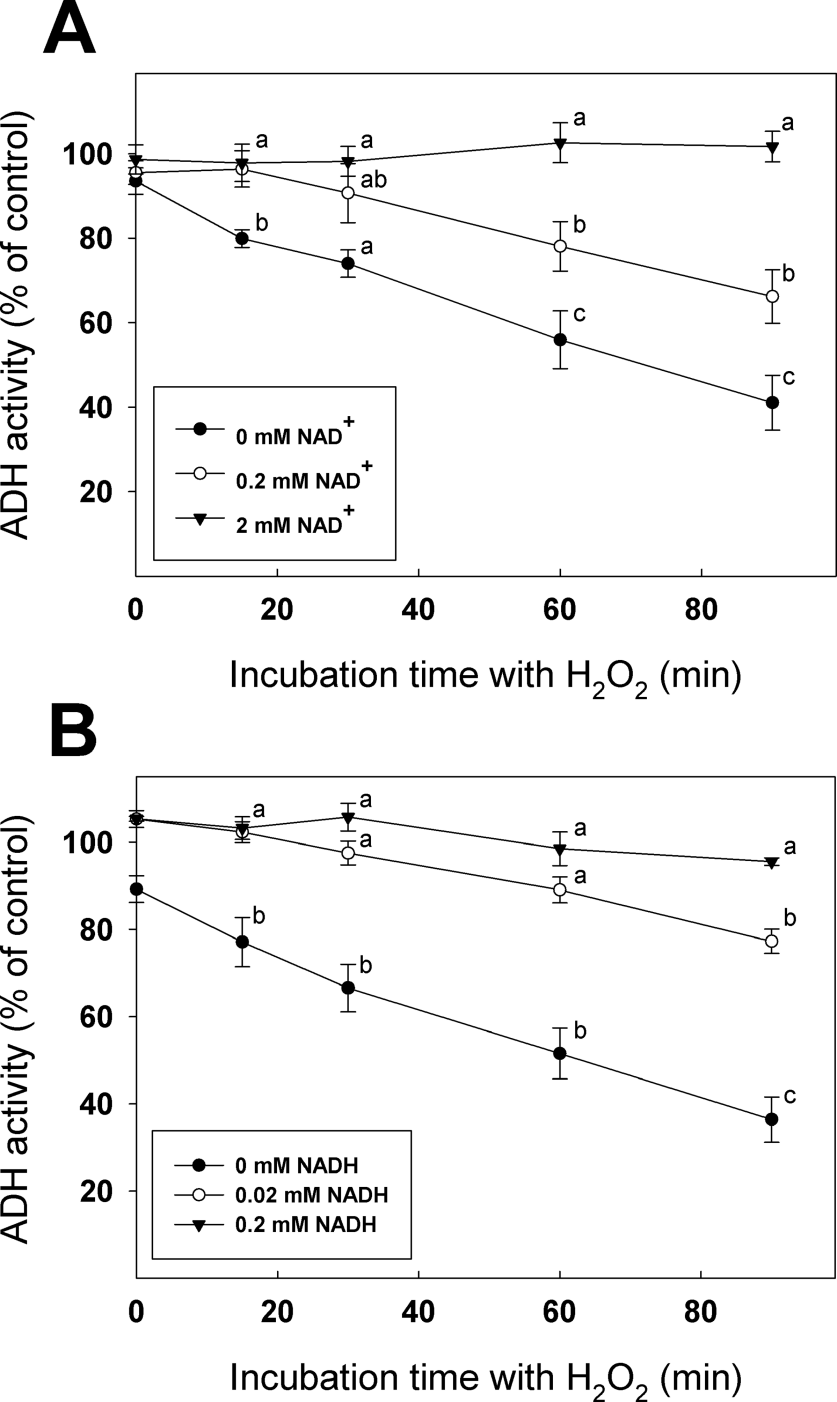
**Protective effect of (A) NAD+ or (B) NADH incubation on ADH treated with H**_**2**_**O**_**2**_. ADH activity was measured at different time points. Data with different letters are significantly different (Student’s *t*-test, *P* < 0.05).

**Fig 6:**
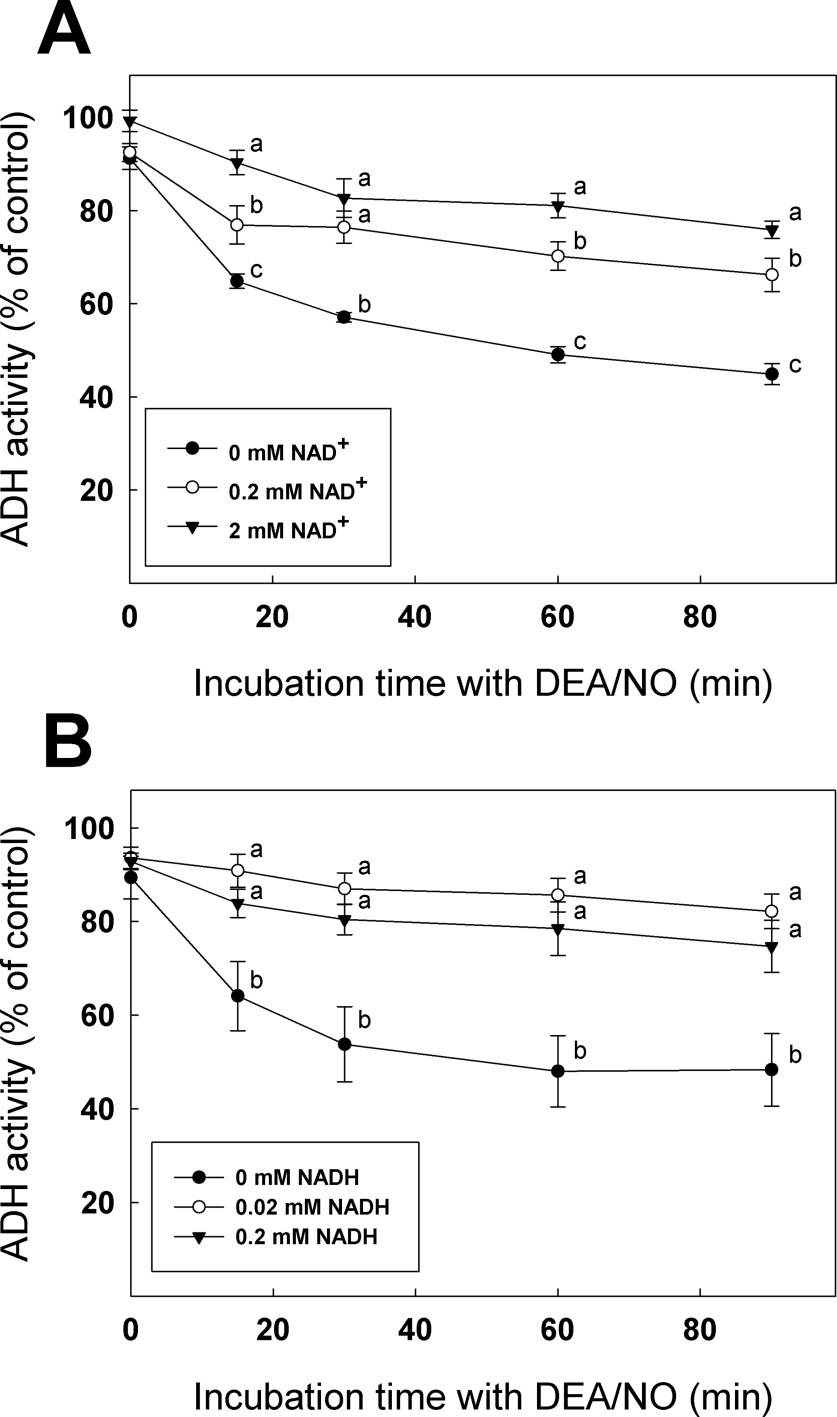
**Protective effect of (A) NAD+ or (B) NADH incubation on ADH treated with DEA/NO.** ADH activity was measured at different time points. Data with different letters are significantly different (Student’s *t*-test, *P* < 0.05).

Since Cys47 is involved in cofactor binding [58], we tested if incubation of recombinant ADH with NAD^+^ or NADH could affect availability of its redox-sensitive Cys residues for modification by H_2_O_2_ and DEA/NO (Figs 5 and 6). In these experiments, ADH activity was measured as a function of their respective control (same NAD^+^ or NADH concentration, but without H_2_O_2_ or DEA/NO). For these experiments, we used a lower concentration of NADH than NAD^+^ because we found that NADH was more effective for protection of ADH. Inhibition of ADH by H_2_O_2_ measured in the presence of 0, 0.2 or 2 mM NAD^+^ revealed that incubation with NAD^+^ reduced significantly the enzyme’s sensitivity to inhibition (Fig 5A). Incubation of recombinant ADH with 0, 0.02 or 0.2 mM NADH also demonstrated that the presence of NADH allows the enzyme to keep a significantly higher activity level in presence of H_2_O_2_ (Fig 5B). Similar experiments were conducted to evaluate ADH sensitivity to DEA/NO in the presence of NAD^+^ and NADH (Fig 6). The remaining activity of the samples containing 0.2 and 2 mM NAD^+^ was significantly higher than the control containing 0 mM NAD^+^(Fig 6A). When the experiment was performed with NADH, (Fig 6B), incubations with 0.02 and 0.2 mM NADH showed significantly higher activity than the control. We also incubated recombinant ADH with 20 mM ethanol and did not find any evidence of a protective affect of ethanol from H_2_O_2_ or DEA/NO, as the samples with or without ethanol were not significantly different (Figure I in S1 File**)**.

### Analysis of ADH C47S and C243S mutants

To study the importance of Cys47 and Cys243 identified above as redox active residues, we produced Cys to Ser mutants and generated the C47S and C243S proteins from the WT *A*. *thaliana* ADH. The recombinant mutant proteins were expressed in *E*. *coli* and purified to apparent electrophoretic homogeneity (Figure J in S1 File). Mutation of Cys47 caused an almost total (> 99.9%) loss of enzyme activity compared to the WT (Table 1). We also found some differences in the calculated specific activity, *k*_cat_ and *K*_m_ values between the C243S mutant and the WT protein. However, the *k*_cat_/*K*_m_ values were not different between both proteins meaning that mutation on Cys243 did not significantly affect ADH kinetic efficiency. It was not possible to determine the *K*_m_ for the C47S mutant due to its extremely low activity.

**Table 1:** Kinetic analysis of recombinant WT and mutant ADH proteins.

Protein	Specific activity (μmol min^-1^ mg^-1^)	*k*_cat_ (s^-1^)	*K*_m_ (mM)	*k*_cat_/*K*_m_ (x 10^3^ M^-1^ S^-1^)
ADH WT	42.7 ± 3.7^a^	31.5 ± 2.7^a^	0.166 ± 0.017^a^	191 ± 8
ADH C47S	0.0054 ± 0.0002^b^	0.0040 ± 0.0001^b^	nd	nd
ADH C243S	70.8 ± 4.0^c^	52.1 ± 3.0^c^	0.251 ± 0.017^b^	208 ± 5

The different superscript letters (a, b, c) in each column indicate that the values are significantly different (Student’s *t*-test, *P*<005). nd: not determined due to lack of significant activity

We obtained fluorescence emission spectra for each protein and used them to generate differential spectra of the mutant proteins relative to the WT (Figure K in S1 File). The spectra for C47S and the WT were very similar, suggesting that the mutation of Cys47 did not disrupt significantly the structure of the enzyme. More dissimilar spectra were obtained when comparing the C243S and WT proteins. To verify the importance of Cys243 during ADH inhibition, we tested if the C243S mutant was sensitive to H_2_O_2_ or DEA/NO (Figure L in S1 File) since, unlike C47S, this ADH mutant was active. Moreover, Cys243 had previously been identified as an *S*-nitrosylation site in *A*. *thaliana* [25]. We observed that incubation of this enzyme with both reagents led to a loss of activity that was similar to the WT, indicating that the C243S mutation did not significantly affect the sensitivity of ADH activity to neither H_2_O_2_ nor DEA/NO.

## Discussion

In this work, we studied the sensitivity of *A*. *thaliana* ADH to different oxidative treatments. We found that the activity of the recombinant enzyme was sensitive to H_2_O_2_ and DEA/NO but was not inhibited by GSSG or GSH + diamide treatments. Incubation of ADH with its cofactors NAD^+^ or NADH could protect to a certain level against inhibition by H_2_O_2_ or DEA/NO. Cys47 and Cys243 were identified as redox active residues that can form mixed disulfide with glutathione. Few site-directed mutants of ADH from all organisms have been described in the literature. Our work provides a first insight into the importance of these Cys residues through the analysis of Cys47S and Cys243S ADH mutants. The mutant lacking the strictly conserved Cys47 had almost no enzyme activity (< 0.1% of the WT enzyme) while the Cys243S mutant had a higher specific activity but similar catalytic efficiency (*k*_cat_/*K*_m_) when compared to WT.

Addition of H_2_O_2_ or diamide to cell culture has been used in literature to induce oxidative stress. Treatment of *A*. *thaliana* suspension cell culture with 88 mM H_2_O_2_ for 16 h has been shown to decrease growth rate without any significant decrease in cell viability [59]. Lower concentrations (10 mM, used in this study) of H_2_O_2_ have also been used to induce oxidative stress in *A*. *thaliana* cells [60]. In *Chlamydomonas reinhardtii*, diamide treatment was used to induce oxidative stress in order to identify *S*-thiolation targets [50]. With the concentrations used in this work, we showed that incubation of *A*. *thaliana* suspension cell culture with diamide had a larger impact on free thiol content than incubation with H_2_O_2_ (Fig 1A). The diamide treatment also had a greater impact on GAPDH activity, an enzyme known to be inactivated by several redox PTMs [61], and used as a control for the effectiveness of the treatments in our experiments. Our data show that ADH activity was exclusively reduced in cells treated with H_2_O_2_ and unaffected by diamide treatment (Fig 1B). This could be explained by the fact that H_2_O_2_ and diamide both induce oxidative stress but by different mechanisms. H_2_O_2_ is well known to induce stress by oxidizing Cys thiol groups to sulphenic acid [60], while diamide reacts rapidly with LMW thiols in cells and promotes protein *S-*thiolation without a requirement for Cys sulfenation [62, 63]. We observed that H_2_O_2_-induced oxidation of recombinant ADH from *A*. *thaliana* decreased enzyme activity (Fig 2A) while *S*-glutathionylation by GSSG or diamide + GSH did not negatively affect the enzyme (Fig 3). Moreover, H_2_O_2_ treatment caused loss of Zn from the enzyme structure, while no metal was released in samples treated with GSSG (Figure D in S1 File). These results suggest that ADH activity can be reduced by oxidation but not by GSH *S*-thiolation (*S-*glutathionylation) similarly to what was observed with *A*. *thaliana* cells stressed by H_2_O_2_ or diamide (Fig 1B). The lower band observed on SDS-PAGE for ADH samples treated with H_2_O_2_ (Fig 2C) suggests the possible formation of the disulfide bonds due to oxidation. However, addition of DTT was not effective in restoring enzyme activity after H_2_O_2_ inhibition (Fig 2E). These results imply that the main modification causing the loss of ADH activity seems to be the irreversible sulfenation of Cys residue(s), as previously observed in YADH [31]. *S*-nitrosylation of recombinant ADH by DEA/NO decreased its activity and formation of nitrosothiols on the enzyme could be visualized with the biotin-switch technique (Fig 2B and 2E). Unlike the H_2_O_2_ treatment, inhibition of recombinant ADH by *S*-nitrosylation could be partially reversed by addition of DTT (Fig 2E). Contrary to sulphenic acid formation, protein *S*-nitrosylation is not known to directly undergo further irreversible modification similar to sulphinic and sulphonic acid [64]. Incomplete recovery of enzyme activity by DTT could be explained by the loss of Zn atoms that was also observed after treatment of ADH with DEA/NO (Figure D in S1 File).

While various plant ADHs were identified to be the target of different redox modification in surveys [24, 25, 29, 38], there is little information on the impact of these modifications on the enzyme activity. Here we have shown that *A*. *thaliana* recombinant ADH was irreversibly inactivated by H_2_O_2_ and reversibly inactivated by DEA/NO. Both of these modifications caused loss of Zn from the enzyme. These results are similar to what was observed in yeast and mammals ADHs [27, 30–32]. Surprisingly, modification of recombinant ADH from *A*. *thaliana* by *S*-glutathionylation did not lead to the same effects observed for oxidation and *S-*nitrosylation as the enzyme activity was not negatively affected due to its modification by glutathione (Fig 3). This absence of effect was unexpected since Cys47, one of the two residues modified by *S*-glutathionylation (Fig 4A) was shown to be required for ADH activity (Table 1). A plausible explanation for these results could be that, under the conditions we used, the modification of ADH by glutathione on Cys47 was sub-stoichiometric. *S-*glutathionylation of this residue could be negligible but still be detected by LC-MS/MS due to the high sensitivity of this technique.

Our results differ from what was observed in *K*. *lactis* [28]. In this case, ADH was shown to be inactivated upon incubation with GSSG. The authors suggested that GSSG treatment could induce disulfide bond formation between ADH monomers via Cys278. While *S*. *cerevisiae* has a Cys residue corresponding to Cys278 (Cys276), this residue is not present in *A*. *thaliana* ADH sequence (Figure E in S1 File). Moreover, our results in Fig 3 do not support a similar mechanism in *A*. *thaliana* ADH as that described for *K*. *lactis* ADH.

Due to the stability of protein *S*-glutathionylation, we were able to perform LC-MS/MS on the modified recombinant enzyme and found Cys47 and Cys243 as redox sensitive residues that can form mixed disulfide with glutathione (Fig 4). Cys47 is involved in the NAD^+^/NADH binding site and is implicated in the coordination of the catalytic Zn atom [58]. In *S*. *cerevisiae*, the corresponding Cys residue (Cys43) was found as a target of H_2_O_2_-induced oxidation leading to loss of enzyme activity [26, 31]. Here, the C47S mutation reduced the enzyme activity to <0.1% of the WT (Table 1), suggesting that this residue is of great importance for ADH activity. Incubation of recombinant ADH with NAD^+^ or NADH was shown to interfere with inhibition of the enzyme by H_2_O_2_ and DEA/NO (Figs 5 and 6). We can hypothesize that binding of NAD^+^ or NADH to the ADH structure could protect Cys47 by making the residue less available for redox modification. Substrate protection from redox modifications were observed in mammal ADH as well [27] but also for other enzymes such as GAPDH. Indeed, it was shown that incubation of GAPC1 with 1,3-bisphosphoglycerate or glyceraldehyde 3-phosphate could protect the redox-sensitive catalytic Cys149 from oxidation by H_2_O_2_ and prevent enzyme inhibition [51]. Our data indicate that incubation of ADH with high concentrations of NAD^+^ and NADH could offer almost total protection from H_2_O_2_ (Fig 5) and partial protection from DEA/NO (Fig 6). This suggests that the strictly conserved Cys47 could be a major target of oxidation and *S-*nitrosylation in *A*. *thaliana* ADH.

ADH Cys243 residue has already been found as a target of *S*-nitrosylation in a survey [25]. Cys243 is located at the periphery of the ADH structure and is exposed to the solvent [58]. Our work shows that mutation of Cys243, which is not conserved in photosynthetic organisms (Figure F in S1 File), did not negatively affect ADH activity. Moreover, the C243S mutant was still sensitive to oxidation and *S*-nitrosylation similarly to the WT (Figure L in S1 File), showing that mutation of this residues does not confer any resistance to these redox modifications. Our results then suggest that even if Cys243 could be targeted by redox modifications [25], this Cys residue may not play an important part in redox regulation of ADH.

We were also able to observe that, compared to NAD^+^, smaller concentrations of NADH were able to protect ADH from inhibition by H_2_O_2_ or DEA/NO (Figs 5 and 6). This is important because the low cofactor concentrations used in these experiments are in the physiological range [65]. During hypoxia, NADH levels are known to increase in plant cells, changing the NADH/NAD^+^ ratio [66, 67]. Our results suggest that conditions increasing NADH levels could decrease ADH sensitivity to inhibition by ROS or RNS, thus providing some protection to enzyme activity under hypoxic conditions. Conversely, reduction of NADH levels could result in higher exposure of ADH to damage by ROS or RNS.

In conclusion, ROS and RNS commonly occur in plant cells under normal conditions and the levels of these compounds can increase dramatically upon exposure to stress, including hypoxic conditions. The present work was initiated to investigate the sensitivity of *A*. *thaliana* ADH to oxidative conditions. ADH catalyzes the final step of the ethanol fermentative pathway and is encoded by a single gene in the model plant *A*. *thaliana*. We found that different oxidative treatments resulted in diverse effects on ADH activity. In cell cultures, ADH activity was decreased by H_2_O_2_, but not by the thiol oxidizing reagent diamide known to induce protein *S*-glutathionylation. When recombinant ADH was subjected to treatments with DEA/NO, there was a loss of activity, which could be reversed by DTT. In contrast, H_2_O_2_ led to irreversible loss of activity. Treatments inducing *S*-glutathionylation had no effect on the enzyme activity. Using nanoLC-MS/MS, we were able to identify two redox-sensitive cysteine residues Cys47 and Cys243 as likely targets for oxidative modifications. Furthermore, site-directed mutagenesis enabled us to identify Cys47 as an essential residue for ADH activity. These results indicate that, while ADH is sensitive to ROS and RNS, the nature of the oxidizing compound ultimately determines the effect on the enzyme activity. Our results also demonstrate that physiological concentrations of the cofactors NAD^+^ and NADH can limit the effects of oxidation on ADH, suggesting that variations in NAD^+^ or NADH also affect ADH sensitivity to oxidative stress.

## Supporting information

S1 File Figure A. Purification of His-tagged recombinant ADH.
Figure B. Stability of stored ADH upon dilution and its sensitivity to reductant.
Figure C. ADH treated with H_2_O_2_ is not reactivated by DTT.
Figure D. Loss of Zn atoms from ADH.
Figure E. Alignment of *A*. *thaliana* ADH sequence with ADH from yeasts.
Figure F. Alignment of ADH sequences from different photosynthetic organisms.
Figure G. CID MS/MS fragmentation spectra of two precursor ions corresponding to peptides containing an intrachain disulfide bond between Cys99 and Cys102.
Figure H. CID MS/MS fragmentation spectra of two precursor ions corresponding to peptides containing an intrachain disulfide bond between Cys105 and Cys113, and between Cys173 and Cys177.
Figure I. Inhibition of ADH in presence of ethanol.
Figure J. SDS-PAGE analysis of the purification of His-tagged recombinant ADH mutants.
Figure K. Fluorescence emission difference spectra of the recombinant ADH mutants relative to WT ADH.
Figure L. Sensitivity of C243S ADH mutant to DEA/NO and H_2_O_2._
(DOCX)

## Abbreviations

ADH: Alcohol dehydrogenase
BioGEE: biotinylated glutathione ethyl ester
BioGSSG: biotinylated glutathione oxidized
GSH: glutathione
GSSG: glutathione oxidized
Cys: cysteine
DEA/NO: diethylamine NONOate
H_2_O_2_: hydrogen peroxide
LMW: low molecular weight
NO: nitric oxide
ONOO^-^: peroxynitrite
PTMs: post-translational modifications
ROS: reactive oxygen species
RNS: reactive nitrogen species
YADH: yeast alcohol dehydrogenase
