# Supplementary material for: Arabidopsis thaliana alcohol dehydrogenase is differently affected by several redox modifications
Source: PLoS One. 2018 Sep 25;13(9):e0204530. doi: 10.1371/journal.pone.0204530 (PMC6155552; doi:10.1371/journal.pone.0204530)
Supplement: S1 File — Figure A. Purification of His-tagged recombinant ADH. Figure B. Stability of stored ADH upon dilution and its sensitivity to reductant. Figure C. ADH treated with H2O2 is not reactivated by DTT. Figure D. Loss of Zn atoms from ADH. Figure E. Alignment of A. thaliana ADH sequence with ADH from yeasts. Figure F. Alignment of ADH sequences from different photosynthetic organisms. Figure G. CID MS/MS fragmentation spectra of two precursor ions corresponding to peptides containing an intrachain disulfide bond between Cys99 and Cys102. Figure H. CID MS/MS fragmentation spectra of two precursor ions corresponding to peptides containing an intrachain disulfide bond between Cys105 and Cys113, and between Cys173 and Cys177. Figure I. Inhibition of ADH in presence of ethanol. Figure J. SDS-PAGE analysis of the purification of His-tagged recombinant ADH mutants. Figure K. Fluorescence emission difference spectra of the recombinant ADH mutants relative to WT ADH. Figure L. Sensitivity of C243S ADH mutant to DEA/NO and H2O2. (DOCX) [file pone.0204530.s001.docx]

Supplementary file to: *Arabidopsis thaliana* alcohol dehydrogenase is differently affected by several redox modifications

**Authors:** Sébastien Dumont^1^, Natalia V. Bykova^2^, Alexia Khaou^1^, Yasmine Besserour^1^, Maude Dorval^1^, and Jean Rivoal^1^

^1^Institut de Recherche en Biologie Végétale, Université de Montréal, Montréal, Québec, Canada

^2^Morden Research and Development Centre, Agriculture and Agri-Food Canada, Morden, Manitoba, Canada

**List of Supplemental Figures:**

**Figure A. Purification of His-tagged recombinant ADH**

**Figure B. Stability of stored ADH upon dilution and its sensitivity to reductant**

**Figure C. ADH treated with H_2_O_2_ is not reactivated by DTT.**

**Figure D. Loss of Zn atoms from ADH**

**Figure E. Alignment of *A. thaliana* ADH sequence with ADH from yeasts**

**Figure F. Alignment of ADH sequences from different photosynthetic organisms**

**Figure G. CID MS/MS fragmentation spectra of two precursor ions corresponding to a peptide containing an intrachain disulfide bond between Cys99 and Cys102**

**Figure H. CID MS/MS fragmentation spectra of two precursor ions corresponding to peptides containing an intrachain disulfide bond between Cys105 and Cys113, and between Cys173 and Cys177**

**Figure I. Inhibition of ADH in presence of ethanol**

**Figure J. SDS-PAGE analysis of the purification of His-tagged recombinant ADH mutants**

**Figure K. Fluorescence emission difference spectra of the recombinant ADH mutants relative to WT ADH**

**Figure L. Sensitivity of C243S ADH mutant to DEA/NO and H_2_O_2_**

**
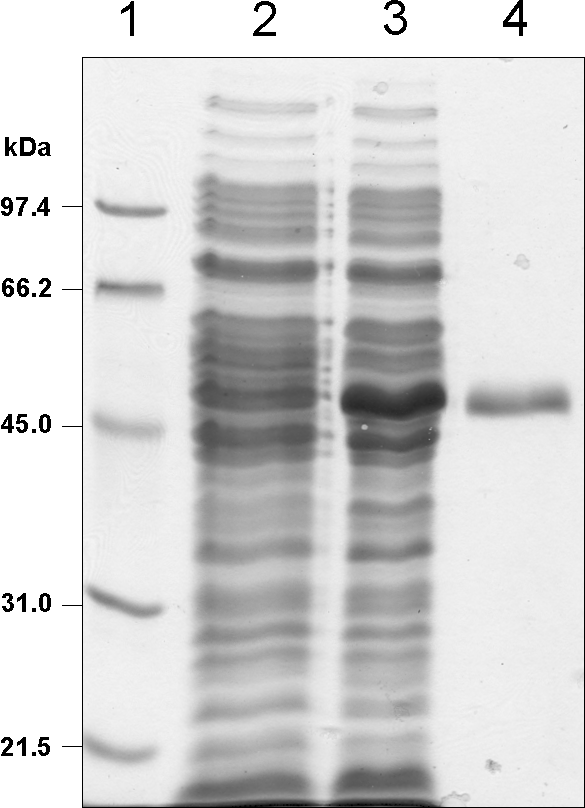
**

**Figure A. Purification of His-tagged recombinant ADH.** SDS-PAGE analysis of recombinant ADH purified from induced *E. coli* cells. Lane 1, molecular weight standards; lane 2, *E. coli* protein extract without induction; lane 3, *E. coli* protein extract after isopropyl β-D-thiogalactoside induction; lane 4, affinity-purified recombinant ADH.

**
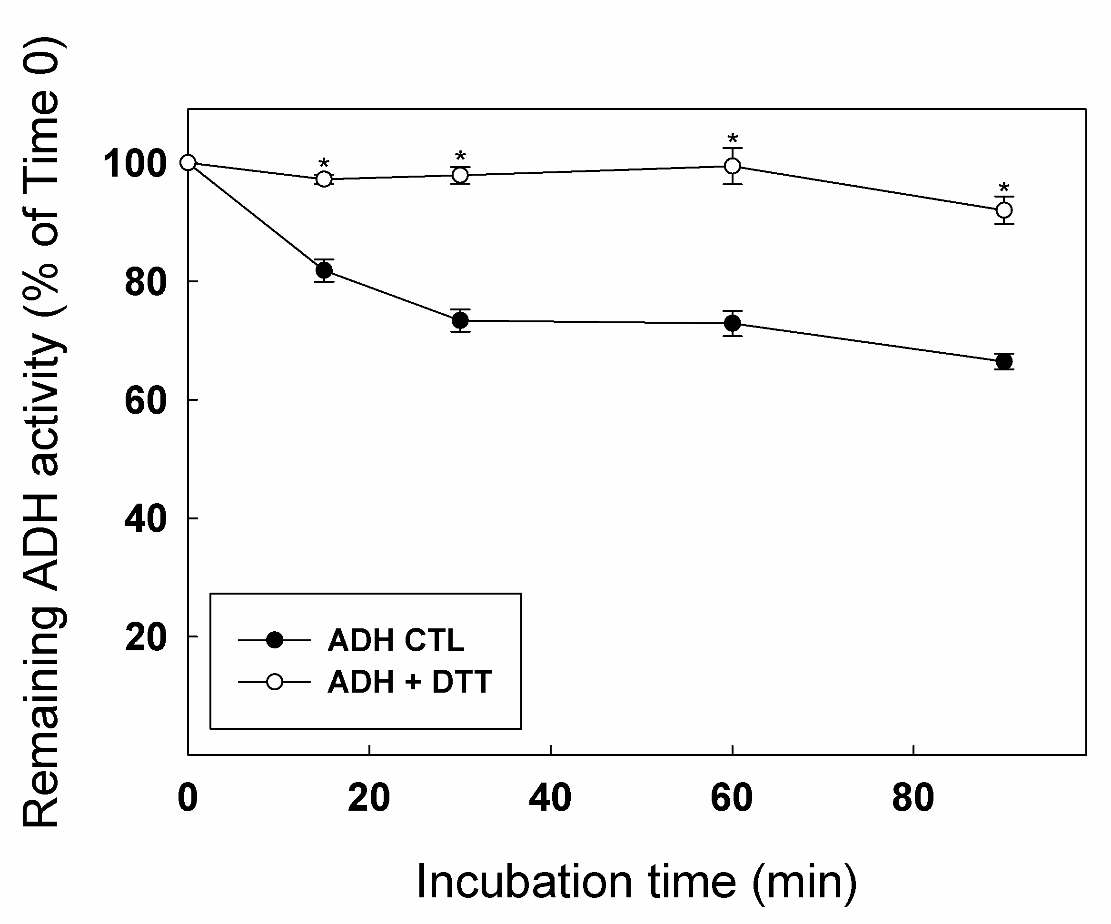
**

**Figure B. Stability of stored ADH upon dilution with or without reductant**. Purified recombinant ADH stored in 50% (v/v) glycerol was diluted to 4 μg/ml in 100 mM Tris-Cl pH 7.5 in the presence or absence of 5 mM DTT. ADH activity was measured at room temperature for different incubation time points. The symbol (*) indicates significant differences for a given time point (Student’s *t-*test, *P* < 0.05).

**
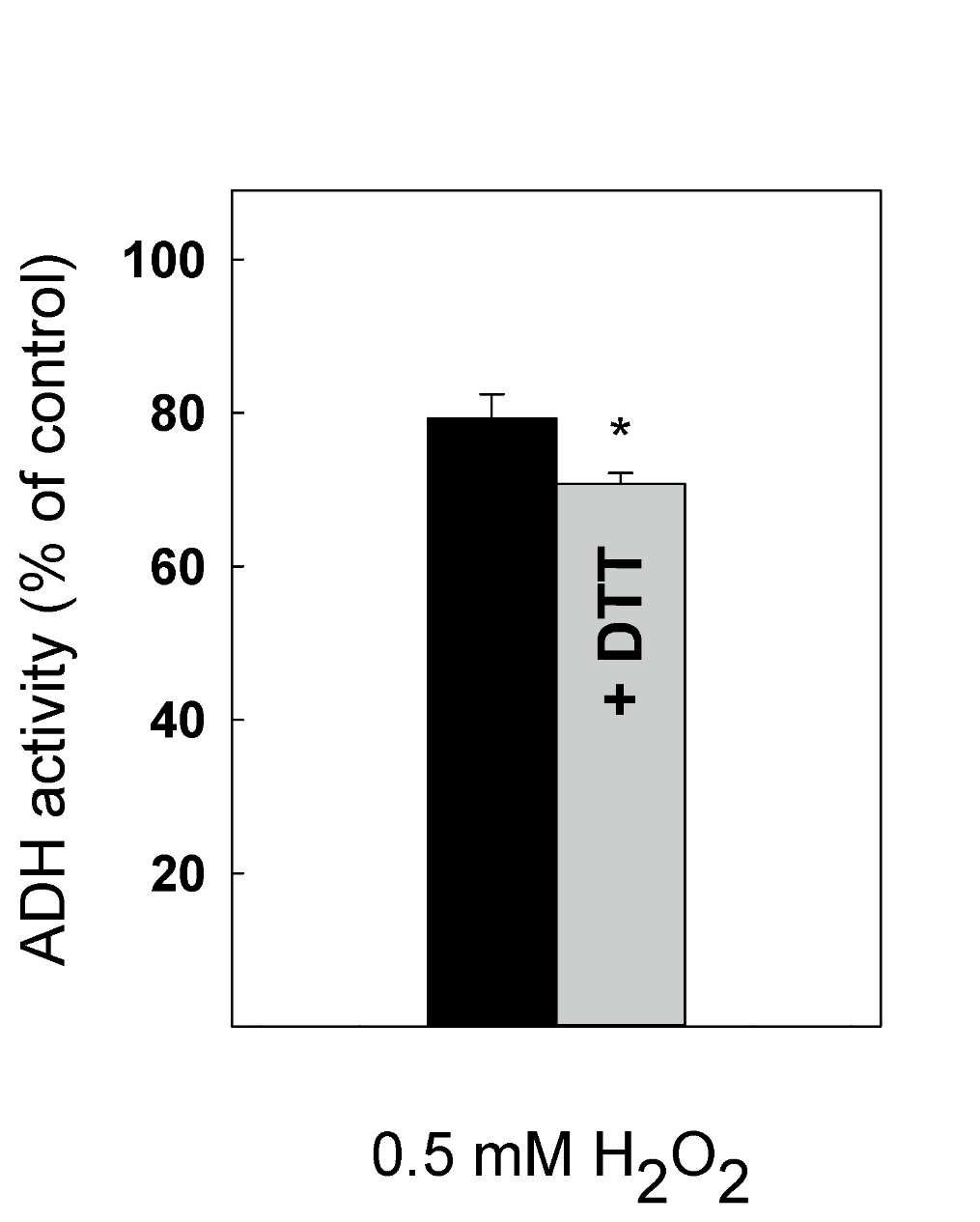
**

**Figure C. ADH treated with H_2_O_2_ is not reactivated by DTT.** ADH was inhibited by 0.5 mM H_2_O_2_ for 30 min, 10 mM DTT was then added in the sample. The symbol (*) indicates a significant difference (Student’s *t*-test, *P* < 0.05)


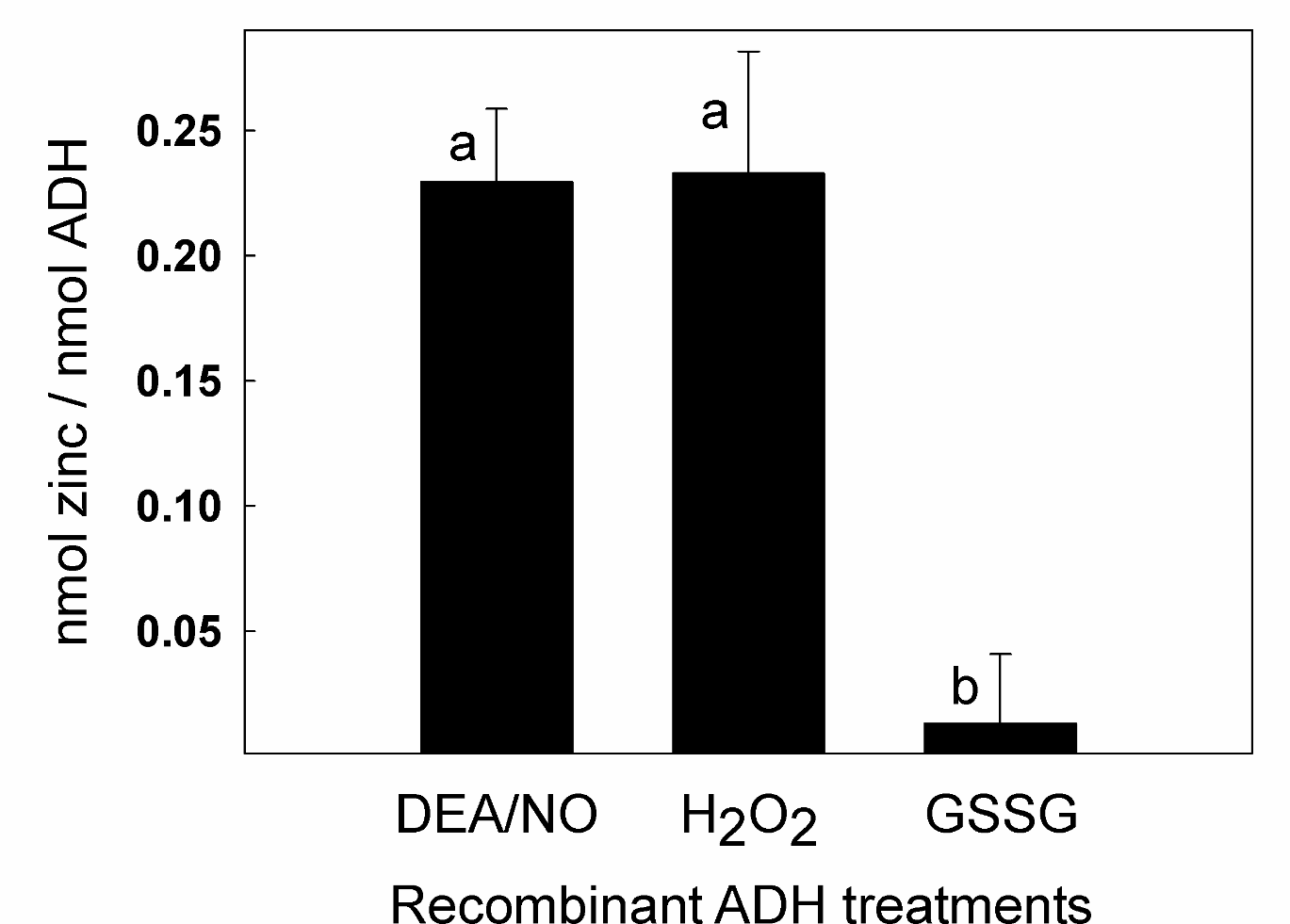


**Figure D. Loss of Zn atoms from ADH.** Recombinant ADH was incubated with DEA/NO, H_2_O_2_ or GSSG. The background value from the control sample was subtracted to the values obtained for each treatment. Different letters indicate significantly different values (Student’s *t*-test, *P* < 0.05).

*** Cys47**

*A. thaliana* MSTTGQIIRCKAAVAWEAGKPLVIEEVEVAPPQKHEVRIKILFTSLCHTDVYFWEAKGQT

*S. cerevisiae* ---MSIPETQKGVIFYESHGKLEYKDIPVPKPKANELLINVKYSGVCHTDLHAWHGDWPL

*K. lactis* -MAASIPETQKGVIFYENGGELQYKDIPVPKPKANELLINVKYSGVCHTDLHAWKGDWPL

*A. thaliana* PL-FPRIFGHEAGGIVESVGEGVTDLQPGDHV-LPIFTGECGECRHCHSEESNMCDLLRI

*S. cerevisiae* PVKLPLVGGHEGAGVVVGMGENVKGWKIGDYAGIKWLNGSCMACEYCELGNESNCPHADL

*K. lactis* PTKLPLVGGHEGAGVVVAMGENVKGWKIGDFAGIKWLNGSCMSCEYCELSNESNCPEADL

*A. thaliana* NTERGGMIHDGESRFSINGKPIYHFLGTSTFSEYTVVHSGQVAKINPDAPLDKVCIVSCG

*S. cerevisiae* ----SGYTHDG------------------SFQQYATADAVQAAHIPQGTDLAQVAPILCA

*K. lactis* ----SGYTHDG------------------SFQQYATADAVQAAKIPVGTDLAEVAPVLCA

*A. thaliana* LSTGLGATLNVAKPKKGQSVAIFG----LGAVGLGAAEGARIAGASRIIGVDFNSKRFDQ

*S. cerevisiae* GITVY-KALKSANLMAGHWVAISGAAGGLGSLAVQYA----KAMGYRVLGIDGGEGKEEL

*K. lactis* GVTVY-KALKSANLKAGDWVAISGAAGGLGSLAVQYA----KAMGYRVLGIDAGEEKAKL

*** Cys243**

*A. thaliana* AKEFGVTECVNPKDHDKPIQQVIAEMTDGGVDRSVECTGSVQAMIQAFECVHDGWGVAVL

*S. cerevisiae* FRSIGGEVFID-FTKEKDIVGAVLKATDGGAHGVINVSVSEAAI-EASTRYVRANGTTVL

*K. lactis* FKDLGGEYFID-FTKSKNIPEEVIEATKGGAHGVINVSVSEFAI-EQSTNYVRSNGTVVL

*A. thaliana* VGVPSKDDAFKTHPMNFLNERTLKGTFFGNYKPKTDIPGVVEKYMNKELELEKFITHTVP

*S. cerevisiae* VGMPAGAKCCSDVFNQVVKSISIVGSYVGNRADTREALDF----FAR--GLVKSPIKVVG

*K. lactis* VGLPRDAKCKSDVFNQVVKSISIVGSYVGNRADTREAIDF----FSR--GLVKAPIHVVG

*** Cys278**

*A. thaliana* FSEINKAFDYMLKGESIRCIITMGA- 379

*S. cerevisiae* LSTLPEIYEKMEKGQIVGRYVVDTSK 348

*K. lactis* LSELPSIYEKMEKGAIVGRYVVDTSK 350

**Figure E. Alignment of *A. thaliana* ADH sequence with ADH from yeasts.** Sequences were obtained from the NCBI resource centre. The sequences were aligned with Clustal W. Cys residues of interest are indicated. Sequence accession numbers are: *Arabidopsis thaliana*, NP_177837.1; *Saccharomyces cerevisiae*, NP_014555.1; *Kluyveromyces lactis*, XP_456023.1.

*A.thaliana* -MSTTGQIIR CKAAVAWEAG KPLVIEEVEV APPQKHEVRI KILFTSLCHT

*** Cys47**

*S.tuberosum* MSTTVGQVIR CKAAVAWEAG KPLVMEEVDV APPQKMEVRL KILYTSLCHT

*Z.mays* -MATAGKVIK CKAAVAWEAG KPLSIEEVEV APPQAMEVRV KILFTSLCHT

*O.sativa* -MATAGKVIK CKAAVAWEAA KPLVIEEVEV APPQAMEVRV KILFTSLCHT

*P.patens* -MSTEGQVIT CKAAIAWEAK KPLSIEDVQV APPQAGEVRI KITHTALCHT

*E.gracilis* ---------- MKAAVVEQFG KPLAIREVPV PEPGYGQVLI KIIASGVCHT

*A.thaliana* DVYFWEAKGQ T-PLFPRIFG HEAGGIVESV GEGVTDLQPG DHVLPIFTG-

*S.tuberosum* DVYFWEAKGQ N-PVFPRILG HEAAGIVESV GEGVTELAPG DHVLPVFTG-

*Z.mays* DVYFWEAKGQ T-PVFPRIFG HEAGGIIESV GEGVTDVAPG DHVLPVFTG-

*O.sativa* DVYFWEAKGQ T-PVFPRIFG HEAGGIVESV GEGVTDLAPG DHVLPVFTG-

*P.patens* DAYTLDGHDP E-GLFPCILG HEAAGIVESV GEGVTEVKAG DHVIPCYQA-

*E.gracilis* DLHVRDGDWY VKPNLPIIPG HEGAGVVVKV GEGVSTLKVG DRVGSAWLHD

*A.thaliana* ECGECRHCHS EESNMCDLLR INTERGGMIH DGESRFSING KPIYHFLGTS

*S.tuberosum* ECKDCAHCKS EESNMCSLLR INTDRGVMIN DGQSRFSING KPIYHFVGTS

*Z.mays* ECKECAHCKS AESNMCDLLR INTDRGVMIA DGKSRFSING KPIYHFVGTS

*O.sativa*  ECKECAHCKS AESNMCDLLR INTDRGVMIG DGKSRFSING KPIYHFVGTS

*P.patens* ECKECKFCLS GKTNLCGKVR SATGVGLMLS DRKSRFSKDG KVIYHFMGTS

*E.gracilis* SCGHCHYCRA GWETVCGHQA QTG------- ---------- -----FASNG

*A.thaliana* TFSEYTVVHS GQVAKINPDA PLDKVCIVSC GLSTGLGATL NVAKPKKGQS

*S.tuberosum* TFSEYTVVHV GCVAKINPLA PLDKVCVLSC GISTGLGATL NVAKPTKGSS

*Z.mays* TFSEYTVMHV GCVAKINPQA PLDKVCVLSC GYSTGLGASI NVAKPPKGST

*O.sativa* TFSEYTVMHV GCVAKINPAA PLDKVCVLSC GISTGLGATI NVAKPPKGST

*P.patens* TFSEYTVVHA VSVAKVNPAA PLDKICLLGC GIPTGLGAVW NTAKVEKGAN

*E.gracilis* CFAEYSIAEA EYIGVIPDGL SYSQAAPVLC AGVTTYKALK ETEVKPGQWV

*** Cys243**

*A.thaliana* VAIFGLGAVG LGAAEGARIA GASRIIGVDF NSKRFDQAKE FGVTECVNPK

*S.tuberosum* VAIFGLGAVG LAAAEGARIA GASRIIGVDL NASRFEQAKK FGVTEFVNPK

*Z.mays* VAVFGLGAVG LAAAEGARIA GASRIIGVDL NPSRFEEARK FGCTEFVNPK

*O.sativa* VAIFGLGAVG LAAAEGARIA GASRIIGIDL NANRFEEARK FGCTEFVNPK

*P.patens* VAIFGLGTVG LAVAEGAKAA GAARIIGVDI DPSKFDRAKD FGVTETLNPK

*E.gracilis* AILGACGGLG HVGVQYAKAM GMKVCAVDFG EERGNYAMNT LGCRSYVDVK

*A.thaliana* D-HDKPIQQV IAEMTDG-GV DRSVECTGSV QAMIQAFECV HDGWGVAVLV

*S.tuberosum* D-YSKPVQEV IAEMTDG-GV DRSVECTGHI DAMISAFECV HDGWGVAVLV

*Z.mays* D-HNKPVQEV LAEMTNG-GV DRSVECTGNI NAMIQAFECV HDGWGVAVLV

*O.sativa* D-HDKPVQQX XAEMTNG-GV DRSVECTGNI NAMIQAFECV HDGWGVAVLV

*P.patens* D-HKKPTQEV IVEMTDG-GV DYSFDCTGNV HVMRSALECC HKGWGTSVII

*E.gracilis* GRSSEEIVAA VKKACDGEGS HGSVVLAPAL PAFRQGLDML RP---VGTCV

*A.thaliana* GVPSKDDAFK THPMNFLNER -TLKGTFFGN YKPKTDIPGV VEKYMNKELE

*S.tuberosum* GVPHKEAVFK THPMNFLNER -TLKGTFFGN YKPRSDIPSV VEKYMNKELE

*Z.mays* GVPHKDAEFK THPMNFLNER -TLKGTFFGN YKPRTDLPNV VELYMKKELE

*O.sativa*  GVPHKDAEFK THPMNFLNER -TLKGTFFGN YKPRTDLPNV VELYMKKELE

*P.patens* GVAASGQEIS TRPFQLVTGR -VWKGTAFGG FKSRSQVPEL VEKYLKKEIK

*E.gracilis* GIALPPGEFS VDLFSMILHR KTMRGSIVG- -----TRQDL NEALEIAGDG

*A.thaliana* LEKFITHTVP FSEINKAFDY MLKGESIRCI ITMGA--

*S.tuberosum* LEKFITHTLP FAEINKAFDL MLKGEGLRCI ITMED--

*Z.mays* VEKFITHSVP FAEINKAFDL MAKGEGIRCI IRMEN--

*O.sativa* VEKFITHSVP FSEINTAFDL MHKGEGIRCI IRMEN--

*P.patens* VDEYITHNMK LDDINEAFDL LHSGKCLRCV LQLSSL-

*E.gracilis* LVHCTVEERK LEDINTVLED MHAGKIKGRV VLRIANE

**Figure F. Alignment of ADH sequences from different photosynthetic organisms.** Sequences were obtained from NCBI resource centre. Alignments were done with Clustal W. Cys residues of interest are indicated. Sequence accession numbers are: *Arabidopsis thaliana*, NP_177837.1; *Solanum tuberosum*, NP_001275080.1; *Zea mays*, NP_001105409.2; *Oryza sativa*, XP_015616853.1; *Physcomitrella patens*, XP_024393222.1; *Euglena gracilis*, ACI12882.1.

**
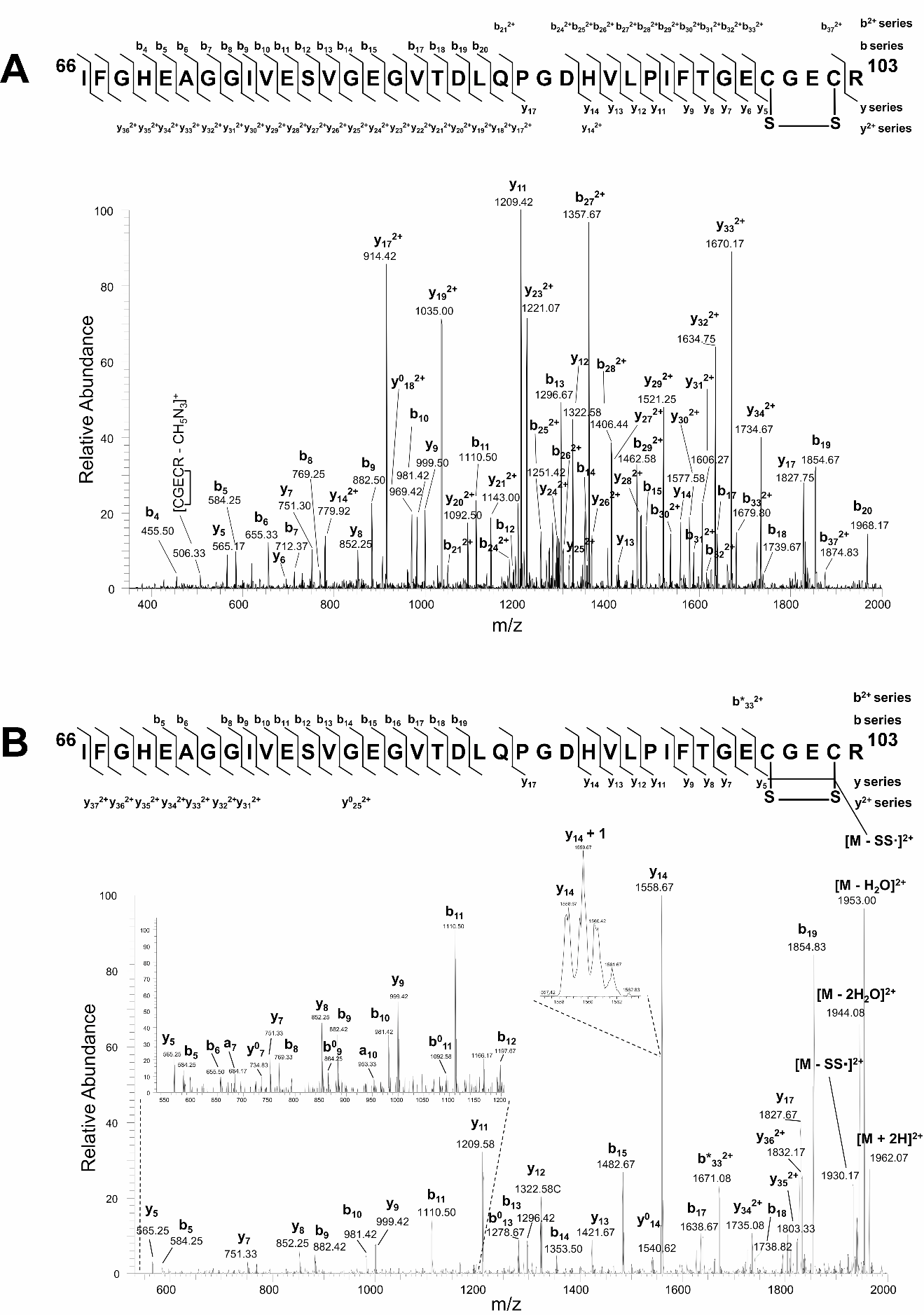
**

**Figure G. CID MS/MS fragmentation spectra of two precursor ions corresponding to a peptide containing an intrachain disulfide** **bond between Cys99 and Cys102.** **(A)** CID MS/MS fragmentation spectrum of the precursor ion at *m/z* of 1962.0663^2+^ corresponding to a peptide with the 38 amino acids long sequence ^66^IFGHEAGGIVESVGEGVTDLQPGDHVLPIFTGECGECR^103^ containing an intrachain disulfide bond. The detection of ion at *m/z* 1930.17^2+^ indicates the loss of perthiyl radical (-SS▪; -64 Da) from the doubly charged precursor resulting in the ion [M - SS▪]^2+^ with the original cysteine residue modified to dehydroalanine. The loss of water (-18 Da) resulted in the ion at m/z 1953.00^2+^ corresponding to [M - H_2_O]^2+^, and the concomitant loss of another water molecule resulted in the ion at m/z 1944.08^2+^ corresponding to [M - 2H_2_O]^2+^. Sequence specific y- and b-type backbone fragment ion signals of different charge states (+1 and +2) identifying the peptide are indicated. The peaks denoted y^0^ and b^*^ are the result of water (-18 Da) or ammonia (-17 Da) loss from the corresponding ion, respectively. A series of singly and doubly charged y-type ions containing the intact intrachain disulfide bond were identified with the mass difference of 2 Da in two oxidised dehydro Cys residues attributed to the presence of cystine residue. Spectral portion with partly isotopically resolved peaks indicating ions charge state is shown in the inset with signature y_n_ + 1 Da (n = 12-14, 17) ions in higher abundance than their corresponding y ions, resulting from the intramolecular hydrogen transfer reactions to the thiyl radical from Val and/or Leu. Experimental and theoretical studies have shown that intramolecular hydrogen transfer to the thiyl radical is a facile process within peptides and cysteine ions [56, 57]. This 66 – 103 aa peptide containing intrachain disulfide bond was identified in GSSG-treated ADH, and it was also present in the control ADH tryptic digest. **(B)** CID MS/MS fragmentation spectrum of the precursor ion at *m/z* of 1308.52^3+^ corresponding to a peptide with the 38 amino acids long sequence ^66^IFGHEAGGIVESVGEGVTDLQPGDHVLPIFTGECGECR^103^ containing an intrachain disulfide bond. In contrast to the fragmentation of the doubly protonated precursor peptide with high intensity ions resulting from the loss of perthiyl radical and one or two water molecules, extensive peptide backbone fragmentation was observed for triply protonated precursor ion under CID conditions. Sequence specific y- and b-type backbone fragment ion signals of different charge states (+1 and +2) identifying the peptide are indicated. The peak denoted y^0^ was the result of water (-18 Da) loss from the corresponding ion. A series of singly and doubly charged y-type ions containing the intact intrachain disulfide bond were identified with the mass difference of 2 Da in two oxidised dehydro Cys residues attributed to the presence of cystine residue. Moreover, the isotopically resolved peaks indicating ions charge state showed normal distribution without y + 1 Da ions in higher abundance than corresponding y ions, indicating CID conditions not optimal for the intramolecular hydrogen transfer reactions in higher charge state precursor ion. The ion at *m/z* of 506.33^+^ corresponds to the internal fragment with the intact disulfide bond that can be attributed to the loss of 59 Da (CH_5_N_3_) from Arg side-chain. This 66 – 103 aa peptide containing intrachain disulfide bond was identified in GSSG-treated ADH, and it was also present in the control ADH tryptic digest.

**
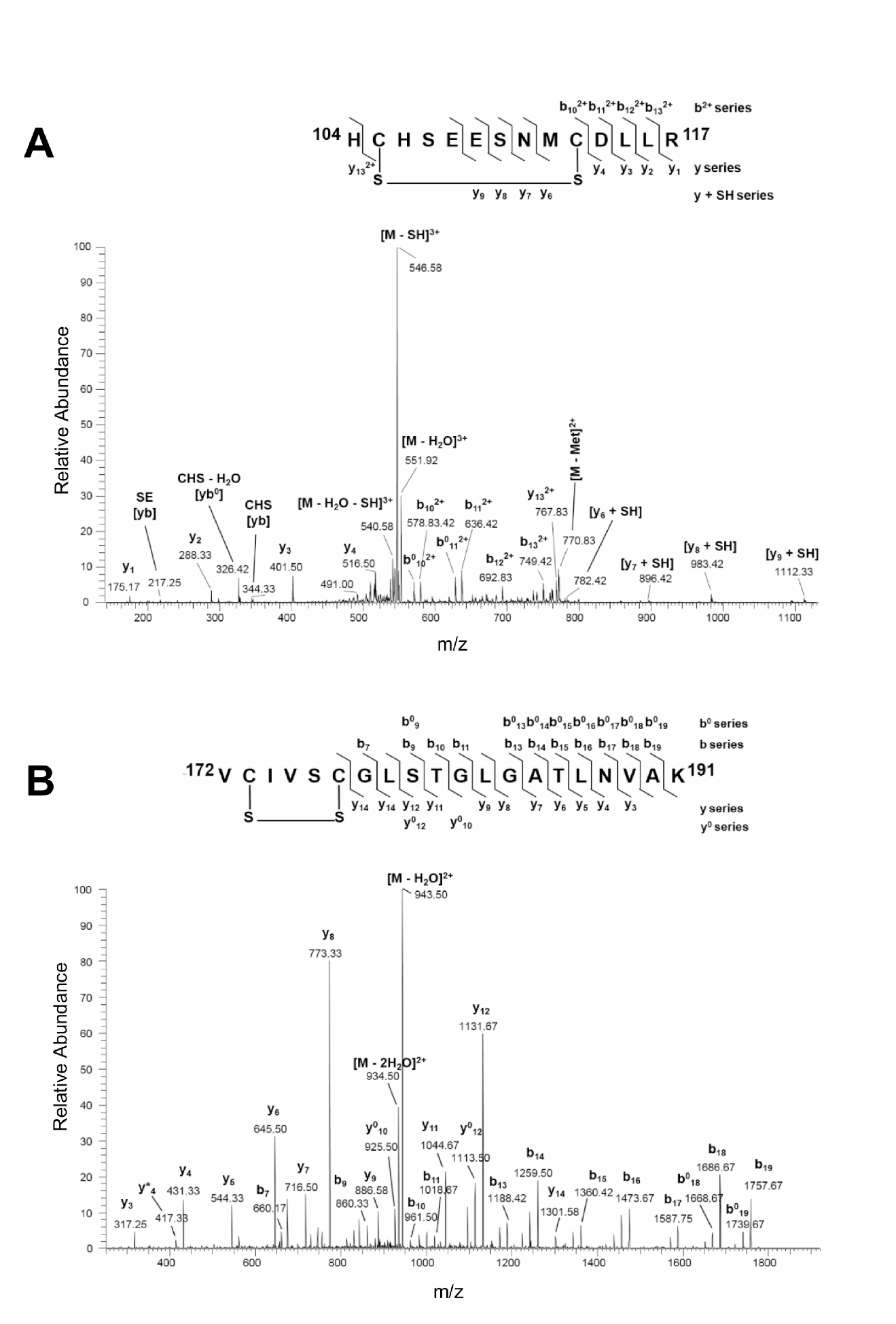
**

**Figure H. CID MS/MS fragmentation spectra of two precursor ions corresponding to peptides containing an intrachain disulfide** **bond between Cys105 and Cys113****, and between Cys173 and Cys177.** **(A)** CID MS/MS fragmentation spectrum of the precursor ion at *m/z* of 557.62^3+^ corresponding to a peptide with the 14 amino acids long sequence ^104^HCHSEESNMCDLLR^117^ containing intrachain disulfide bond. The loss of water (-18 Da) resulted in the ion at m/z 551.92^3+^ corresponding to [M - H_2_O]^3+^ and the loss of methionine residue from the intrachain disulfide-linked peptide ion due to internal fragmentation resulted in the doubly changed ion m/z 770.83^2+^ corresponding to [M - Met]^2+^. The concomitant signature neutral loss of 33 Da (•SH) due to the direct cleavage first at the disulfide bond with subsequent elimination of •SH to form dehydroalanine resulted in the ion at m/z 546.58^3+^ corresponding to [M - SH]^3+^. Sequence specific singly charged y- and b-type backbone fragment ion signals identifying the peptide are indicated. The peaks denoted b^0^ are the result of water (-18 Da) loss from the corresponding ion, yb type ions resulting from internal fragmentation are shown, and y type ions with one cysteine residue being modified to a sulfhydryl group (SH) as a result of disulfide bond cleavage are indicated. A series of doubly charged b-type ions containing the intact intrachain disulfide bond were identified with the mass difference of 2 Da in two oxidised dehydro Cys residues attributed to the presence of cystine residue. This 104 – 117 aa peptide containing intrachain disulfide bond was identified in GSSG-treated ADH, and it was also present in the control ADH tryptic digest. **(B)** CID MS/MS fragmentation spectrum of the precursor ion at *m/z* of 952.57^2+^ corresponding to a peptide with the 20 amino acids long sequence ^172^VCIVSCGLSTGLGATLNVAK^191^ containing intrachain disulfide bond. The loss of water (-18 Da) resulted in the ion at m/z 943.50^2+^ corresponding to [M - H_2_O]^2+^, and the concomitant loss of another water molecule resulted in the ion at m/z 934.50^2+^ corresponding to [M - 2H_2_O]^2+^. Sequence specific singly charged y- and b-type backbone fragment ion signals identifying the peptide are indicated. The peaks denoted y^0^ and b^0^ are the result of water (-18 Da) loss from the corresponding ion. A series of singly charged b-type ions containing the intact intrachain disulfide bond were identified with the mass difference of 2 Da in two oxidised dehydro Cys residues attributed to the presence of cystine residue. This 172 – 191 aa peptide containing intrachain disulfide bond was identified in GSSG-treated ADH, and it was also present in the control ADH tryptic digest.

**

**

**Figure I. Inhibition of ADH in presence of ethanol.** Recombinant ADH was incubated with or without 20 mM ethanol (EtOH) and H_2_O_2_ or DEA/NO was added in the samples. ADH activity was measured at different time points. Student’s *t*-test analysis indicated no significant differences between incubation with or without EtOH.


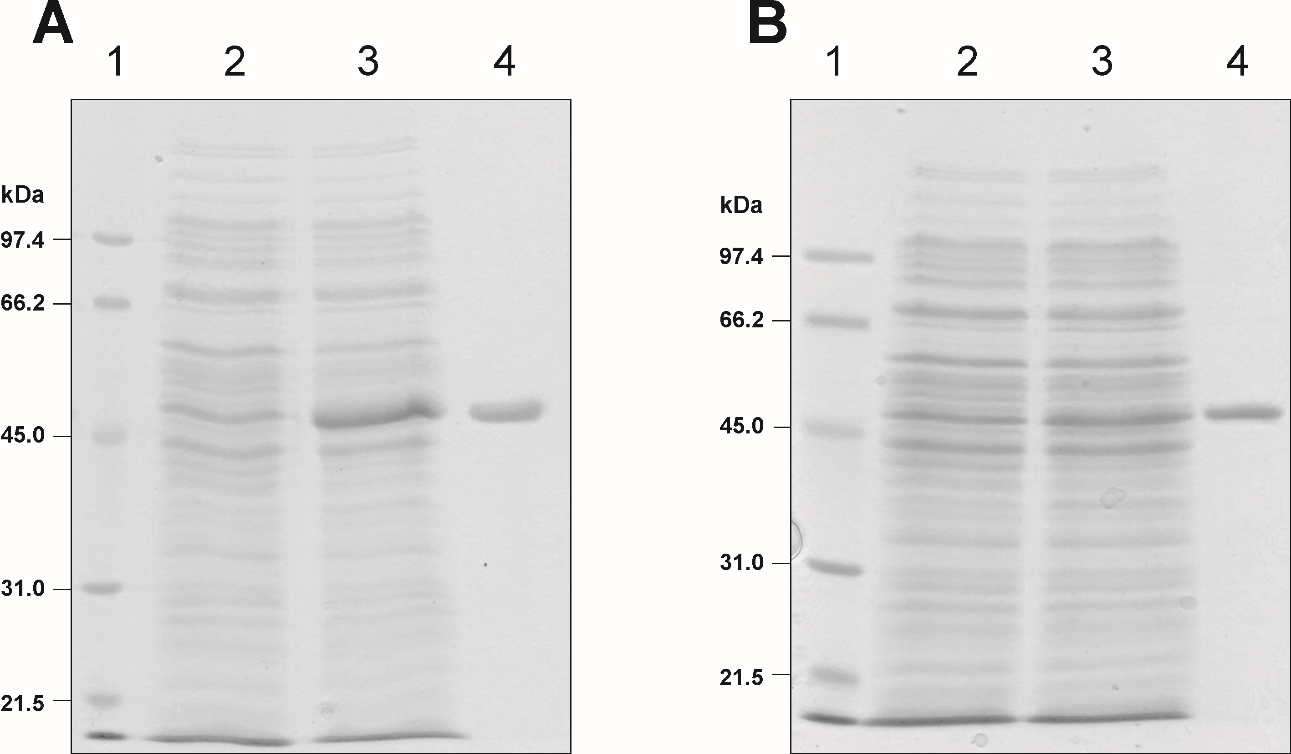


**Figure J. SDS-PAGE analysis of the purification of His-tagged recombinant ADH mutants****.** Purification steps of **(A)** C47S and **(B)** C243S ADH mutants. Lane 1, molecular weight standards; lane 2, *E. coli* protein extract without induction; lane 3, *E. coli* protein extract after isopropyl ß-D-thiogalactoside induction; lane 4, affinity-purified recombinant ADH mutants.





**Figure K. Fluorescence emission difference spectra of the recombinant ADH mutants relative to WT ADH.** Fluorescence emission spectra of different recombinant proteins were recorded using an excitation wavelength of 274 nm. The measured spectrum of WT ADH was subtracted from the mutant spectra.





**Figure L. Sensitivity of C243S ADH mutant to DEA/NO and H_2_O_2._** The effects of H_2_O_2_ and DEA/NO treatments on C243S ADH mutant were evaluated in comparison to WT ADH. Student’s *t*-test analysis indicated no significant difference between WT and C243S for H_2_O_2_ and DEA/NO treatments after 15 min.
